# Aristotle’s Theory of Deviance and Contemporary Symbolic Interactionist Scholarship: Learning from the Past, Extending the Present, and Engaging the Future

**DOI:** 10.1007/s12108-014-9250-9

**Published:** 2015-03-06

**Authors:** Robert Prus

**Affiliations:** University of Waterloo, Waterloo, Canada

**Keywords:** Aristotle, Plato, Deviance, Regulation, Symbolic Interaction, Pragmatism, Knowing and Acting, Nicomachean Ethics, Rhetoric, Ethnography, Character

## Abstract

Although his work has been largely overlooked by symbolic interactionists and other students of deviance, Aristotle (c384-322BCE) addresses community life, activity, agency, and persuasive interchange in ways that not only are remarkably consistent with contemporary symbolic interactionist approaches to deviance, but that also conceptually inform present day theories of deviance and provide valuable transhistorical comparison points for subsequent analysis. Following (1) a brief overview of an interactionist approach to the study of deviance, attention is given to (2) classical Greek conceptions of good and evil (especially as these are articulated by Plato) before turning more directly to (3) Aristotle’s notions of wrongdoing as this is reflected in his considerations of community, morality, agency, and culpability. While informed by Aristotle’s considerations of causality (as addressed in *Physics* and *Metaphysics*), this statement builds most centrally on Aristotle’s *Nicomachean Ethics* and *Rhetoric*. Striving for a broader understanding of deviance as a humanly engaged feature of community life, the paper briefly compares Aristotle’s “theory of deviance” with Prus and Grills (2003) interactionist analysis of deviance. The paper (4) concludes with an assessment of the relative contributions of contemporary interactionist scholarship and Aristotle’s materials for the study of deviance as a community-engaged process.

## Introduction

Men do wrong when they think it can be done and when they think it can be done by them; when they think that their action will either be undiscovered, or if discovered will remain unpunished; or if it is punished that the punishment will be less than the profit to themselves or to those for whom they care. (Aristotle, Rhetoric, BI, xii [Freese, trans.])

It may be tempting to assume that the social sciences and the study of human knowing and acting have made great advances compared to what scholars had developed twenty-five hundred years ago, but a closer examination of the classical Greek literature (c700-300BCE) indicates an exceedingly valuable set of materials that contemporary social scientists have overlooked.

As part of a broader program of study that traces pragmatist thought back to the classical Greek era, this statement considers the particular relevance of Aristotle’s work on deviance for the contemporary social sciences, especially for the sociological theory developed in symbolic interactionism (see Mead 1934; Blumer 1969; Lofland 1976; Strauss 1993; Prus 1996, 1997, 1999; Musolf 1998; Reynolds and Herman 2003; Prus and Grills 2003).

In discussing “Aristotle’s theory of deviance,” it should be recognized that although Aristotle provides an exceptional array of conceptual materials and analytic resources pertaining to the study of deviance as a community-based essence, he does not provide a text entitled, “a theory of deviance.” Indeed, the material presented here is a composite statement primarily developed from Aristotle’s work on ethics (as the study of community-based knowing and acting) and rhetoric (as the study of persuasive interchange and contested reality).

As a further caveat, it may be noted that the term “deviance” (broadly denoting some negatively defined human-quality) appears been derived from the Latin *devia*, referring to “a turning or moving away of some sort.” The term “deviance” may not found in the classical Greek literature, but it is abundantly clear that Homer, Plato, Aristotle and others of the classical Greek and Latin eras were well aware of the negative references to people’s activities, appearances, thoughts, intentions, behavioral outcomes, and reputations, along with the associated matters of deceit, maliciousness, condemnation, sanctions, accusations, defenses, compromises, and shame.

Given the incredible advances made in the physical sciences and associated socially enabling technology, it is easy to assume that the contemporary social sciences, likewise, would be greatly advanced relative to what one encounters in the classical Greek literature. However, despite some particularly important contemporary conceptual developments in the social sciences readers are apt to find much of value in “Aristotle’s theory of deviance.” Indeed, the challenge may be one of absorbing the notably substantial and conceptually sophisticated contributions of Aristotle’s conceptualization of human knowing and acting.

Following (1) a brief overview of the interactionist approach to the study of deviance, this paper gives attention to (2) Greek scholarship and Plato’s considerations of deviance before focusing more directly on (3) Aristotle’s notions of deviance, morality, and identity work. As the ensuing analysis indicates, these materials can be especially helpful in developing transcontextual and transhistorical comparative analyses of human group life as well as more directly enabling set of conceptual formulations.

## An Interactionist Approach to Deviance

Interactionist thought on deviance, like all matters pertaining to human knowing and acting, is to be understood as a social product and a social process; as a socially constituted essence. For instance, after defining deviance as “any activity, actor, idea, or humanly produced situation that some audience defines as threatening, disturbing, offensive, immoral, evil, disreputable or negative in some way,” Prus and Grills (2003) attend to processes of the following sort: defining deviance, identifying deviants, becoming involved in deviance, engaging and sustaining subcultural and solitary deviance, regulating deviance, and experiencing treatment and disinvolvement.

Although the study of deviance as “something in the making” corresponds notably with American pragmatist philosophy (of which interactionism is a sociological derivative), interactionism differs from pragmatist philosophy in insisting on the necessity of examining and testing out conceptual notions in the instances in which human group life takes place. The objective, thus, is to build theory “from the ground up” by continuously examining and assessing notions of theory relative to the ways that people, as purposive agents and interactors, do things in the course of ongoing community life.

This position is articulated particularly effectively by Herbert Blumer (1969). Following George Herbert Mead in developing a theory of human knowing and acting, Blumer also attends to Charles Horton Cooley, Robert Park, Ellsworth Faris, and others who sought to achieve intimate familiarity with their human subject matter through sustained observation and interchange with people about the ways in which those people make sense of and act toward the situation in which they find themselves.

In addition to Blumer’s own published ethnographic ventures (e.g., Blumer 1933; Blumer and Hauser, 1933), Blumer’s emphasis on studying human knowing and acting benefited greatly from other ethnographic works conducted in what would become known as the Chicago tradition.^1^ Relatedly, whereas the term *symbolic interactionism* was initially coined by Herbert Blumer in 1937 (Blumer 1969), it since has been used more loosely to encompass a broader array of approaches (e.g., see Manis and Meltzer 1967; Prus 1996; Reynolds and Herman 2003). Nonetheless, It should be noted that the approach adopted in this paper very much exemplifies Chicago-style or Blumerian symbolic interactionism.

Since both American pragmatist theory and symbolic interactionism so prominently took root in the Midwestern United States, it is understandable that most textbook authors have defined symbolic interactionism and American pragmatism as distinctly American developments, as the unique configurations of a pluralist, democratic society. Thus, it may come as a surprise to some to recognize that Meadian social thought is an extension of the German hermeneutic (interpretive) social theory of Wilhelm Dilthey (1833–1911; see Prus 1996). Still, it may seem even more incredulous to acknowledge the rather substantial development of pragmatist social thought in classical Greek scholarship (c700-300BCE; see Prus 2003; 2004; 2007a; 2008a; 2009; 2011a; 2011b; 2013a; 2013b; 2013c); Puddephatt and Prus 2007; Prus and Burk 2010; Prus and Camara 2010).

Clearly, it is beyond the scope of this paper to address pragmatist thought among the Greeks in more comprehensive terms (in what still survives as a massive and highly sophisticated literature; see, for example, the works of Plato, Aristotle, Isocrates, Thucydides, and Xenophon). More modestly, it will not be possible to even acknowledge all of Aristotle’s work that is relevant to the study of deviance within the confines of this paper.

Not only will a statement on Aristotle’s notions of deviance require the introduction of some contextual background materials on other classical Greek philosophers (particularly Plato), but the set of Aristotle’s writings that pertains to the study of deviance as realms of human knowing and acting also is extensive, multifaceted, and analytically complex.

Thus, before turning to Aristotle’s work more directly, it is important to (a) appreciate some commonly overlooked features of Greek scholarship and (b) acknowledge Plato’s significance for comprehending contemporary notions of deviance. Although this material may seem diversionary in some respects, it is important for understanding contemporary notions of morality and social theory as well as Aristotle’s works on deviance more particularly.

## Classical Greek Scholarship

While Greek scholarship was centrally enabled by the development of a sophisticated phonetic written alphabet (c800BCE; see Martin, 1994), Greek scholars of the classical era (c700-300BCE) should *not* be seen as being of one mind on matters pertaining to philosophy more generally or the consideration of human knowing and acting more specifically. Indeed, a closer examination of their texts reveals a wide (and often conflicting) array of notions pertaining to the nature of the world, human existence, divinity, morality, human agency, process, and the like. It is here, too, that one may begin to appreciate some of the longstanding interlinkages of theology and deviance as well as some of the difficulties that scholars have had in maintaining more secular or pluralist approaches to the study of deviance over the millennia.

Although the poets Homer (c700BCE; *Iliad*, *Odyssey*) and Hesiod (c700BCE; *Theogony, Work and Days*) represent consequential reference points in the development of subsequent Greek texts (and classical studies), the viewpoints that these poets (and the Greek playwrights - Aeschylus, c525-456BCE; Sophocles, c495-405BCE; Euripides, c480-406BCE) present on the Greek gods are given little credibility among Greek philosophers and historians. Indeed, the early Greek scholars adopted an assortment of standpoints that differed dramatically from the images of the worlds of the superheroes and gods (especially the Olympian gods) that commonly are invoked to characterize classical Greek Greek conceptions of divinity.

Thus, for instance, while Protagoras (c480-411BCE) encountered the wrath of some Greeks for refusing to confirm the existence of the gods, Herodotus (484-424BCE; *The Histories*) explicitly denounces the popular Greek gods as the fabrications of Homer and Hesiod and attributes their origin to Egyptian sources. Plato (*Republic, Laws*) also is highly critical of poetic renditions of divinity. Aristotle, in turn, gives little credence to either the gods of the poets or the theological viewpoints of Socrates and Plato.

Reviewing Greek (and Roman) philosophic positions on divinity, Cicero (106-43BCE; *On the Nature of the Gods*) provides a compact but extended review of about 30 conceptions of divinity (as in variants of theism and atheism), each of which offer notably different viewpoints on divinity morality, agency, and culpability (as in deviance). Still, of the early Greek standpoints on religion and morality, it is Plato (who follows Pythagoras and Socrates) and Aristotle whose works are especially relevant to contemporary considerations of theology and deviance.

## Acknowledging Plato

Although often dismissed as an idealist, Plato merits extended attention from social scientists for both the relevance of the moralist and theological materials he develops for contemporary conceptions of deviance in western society and his broader, often pragmatist oriented considerations of human group life.

Thus, beyond any impact Plato may have had as a moralist and theologian in his own time (as a proponent of the theology promoted by Socrates [c469-399BCE] and Pythagoras [c580-500BCE]), Plato appears have been pivotal in shaping Western religion and morality. Clearly predating Christian and Islamic theology, the religious texts, (especially *Timaeus* and *Phaedo*) that Plato develops are highly consistent with much that later would be recorded as belonging to the Jews, Christians, and Islamics.

Without engaging these affinities more fully at present, it may be observed that many of Plato’s texts not only reflect religiously-inspired notions of deviance, but the broader notions of good and evil that characterize Western images of morality and deviance, also resonate strongly with Plato’s work.

Those familiar with Plato’s texts will quickly observe that Plato’s scholarship extends well beyond his theological viewpoints and that the theologians who followed Plato disregarded much of Plato’s more scholarly (“pagan”) statements, choosing to focus more exclusively on Plato’s materials that dealt with divinity and ways of fostering what Augustine (c354-430) would term *The City of God*.

In addition to his extended relevance for understanding conceptions of Western religions and associated notions of deviance, Plato also may be envisioned as a utopian (socialist) philosopher,^2^ a moral entrepreneur and policy maker, a conceptual idealist, a dialectician, and a pragmatist philosopher.^3^ As might be inferred, Plato’s works contain a series of unresolved tensions. At a broader scholarly (sociology of knowledge) level, Plato may be best appreciated for the incredible diversity of thought he introduces and his insistence on dialectic (i.e., sustained comparative) analysis.

For our more immediate purposes, the “deviants” (as evidenced by evil, immorality, sin, lawlessness) that Plato (see *Republic, Laws*) identifies are people who variously (a) fail to respect divinity, (b) detract from the common good, and (c) disrupt the social order of the community. While often insisting on a divinely enabled, as well as a more universal sense of morality, Plato also explicitly acknowledges the *definitional relativism* of both laws and people’s notions of wrongdoing across human communities and the more particular groupings of people within.^4^

The sources or explanations of deviance and the remedial measures that Plato identifies also are rather diverse. As a theologian following Socrates, Plato argues that people do not intend to pursue deviance but are led astray by sensate desires, weak wills, and unsavory influences. However, there is the hope (with the help of divinely enabled philosophers) that most may be shown ways of living more virtuous life-styles.

Plato further argues that greed, disregard of traditions, and personal quests for independence threatens community life more generally. Remedies are to be achieved through extended education, philosophically trained guardians, constitutional laws, and correctional facilities. Likewise, censorship is to be practiced extensively, with poets and other evil influences to be banished from the community.

*In more pragmatist terms* Plato also recognizes the variability of community definitions of deviance and morality, the influences of people’s companions on their thoughts and activities, the necessity of human instruction and learning processes, and the deliberative (reflective) gains that people may associate with wrongdoing as well as the ways in which wrongdoers may exempt themselves from religious and secular moralities.^5^

While often presenting contrasting viewpoints in extended detail and in comparative, analytic terms in the course of developing his dialogues, Plato may be best known for the Socratic emphasis on promoting divinely-inspired truth, justice, and wisdom on both community and individual levels. Relatedly, as followers of Socrates, philosophers are encouraged to provide the essential theological and moral guidelines for human conduct that would enable people to achieve more viable after-life states.^6^ Still, there is much in Plato that has a pragmatist quality and his student Aristotle appears to have made the pragmatist emphasis on the nature of human knowing and acting the centralizing feature of his more distinctively secular scholarship.

## Aristotle on Human Knowing and Acting

Although Plato is often characterized as an “idealist” and Aristotle as an “objectivist,” these definitions (as already indicated for Plato) are only partially accurate at best. Still, Aristotle’s approach to human knowing and acting is notably different from that of Plato and it is extremely important to acknowledge their relative similarities and differences.^7^

First and foremost, Aristotle is *not* a theologian. While he invokes the notion of a prime mover (*Physics*; *Metaphysics*) to account for existence on a philosophic level, Aristotle also observes that the source [God] would be unlike anything we could imagine. Relatedly, rather than emphasizing knowing and acting as the a means of achieving oneness with divinity, Aristotle focuses on knowing and acting as the fundamental and enabling matter of the human condition.

Like Plato, Aristotle makes reference to the *psyche* (Greek) – often translated as *de anima* (Latin), *geiste* (German), and *mind* and *soul* (English). However, in contrast to Plato who sometimes subscribes to a divinely-based soul, Aristotle makes no spiritual claims whatsoever about the life-energy that he associates with *all* living entities (humans, other animals, and plants).^8^ For, Aristotle, as well, there is no psyche-body dualism. Living is an embodied process characterized by variable creature capacities for sensation and motion.

More specifically, although humans differ from other animals by virtue of biological features (as all specific species of animals also differ from others), it is people who most notably possess capacities for speech, recollectable memories, deliberation (group and individual reflectivity), and meaningful, intentioned activity. Aristotle need not have been the very first Greek philosopher to describe people as *social animals*, but he is emphatic on this matter.^9^

Likewise, whereas Plato (as a theologian) at times dismisses the sensate or material world as unreal or illusionary and often debates whether people can truly know the things of the sensate world, Aristotle insists on attending to the humanly known and engaged world as the *primary reality*.

In a manner that also displays Plato’s influence, Aristotle continues to stress civil morality and individual virtues. Although envisioning Plato’s versions of a socialist state (*Republic* and *Laws*) as untenable, Aristotle’s emphasis is on fostering a pluralist society (informed by secular scholarship) that is intended to enable people to attain the best that can be humanly achieved. Like Plato, Aristotle places great value on human virtues (as in justice, wisdom, courage, self regulation, truthfulness, and prudence). However, in contrast to Plato who at times finds these matters unattainable in human terms, Aristotle approaches virtues totally as realms of human capacities, activities, and involvements. Aristotle very much focuses, in pragmatist terms, on “what is” and how one may best conceptualize the actualities of human knowing and acting.

Still, Aristotle (like Plato) intends to find ways of improving things for people at both community and individual levels. Thus, on occasion, Aristotle’s quest for generating more virtuous human life-worlds and practices tends to obscure and detract from his more scholarly, secular analysis of the human condition.

Despite the challenges of disentangling some prescriptive emphases from pragmatist emphases and comparative analyses as these pertain to the human condition, it is important that social scientists not let these other matters divert them from attending more carefully to the remarkable conceptual materials that both Plato and Aristotle develop. Not only do they attempt to define their terms of reference in instructive analytical terms, but they also engage these matters in sustained, comparative (dialectic) manners.

Still, another important difference between Plato and Aristotle revolves around their notions of concepts. While Plato (in many of his dialogues) works with ideal forms that appear only as rough approximations in the humanly known or sensate world (*Republic* and *Laws* are notable exceptions in this regard), Aristotle insists that people’s concepts of things are developed from familiarity with and examinations of the *instances* of things that people encounter in the sensate world and assess in comparative terms (differences, similarities, and implications). Aristotle is clearly aware of the value of deductive logic but he sees analytic induction (comparative analysis) as foundational to all knowing. The task, for Aristotle, is to develop concepts that allow people to better know the things they encounter in the sensate world.

To set the stage for those more familiar with interactionist scholarship, we may observe that Aristotle, like George Herbert Mead (1934) and Herbert Blumer (1969), is fundamentally concerned with the enacted nature of human group life. Like Mead and Blumer, as well, Aristotle envisions deviance *not* as something that requires a special theory or explanation but as something that is the natural product of human association.

Deviance, accordingly, is to be explained as activity in the most fundamental of humanly engaged terms. Thus, although there are things that Aristotle explicitly defines as evil in reference to community standards or things that he envisions as good and bad for individuals and the communities in which they live, his approach to *the study* of matters pertaining to human knowing and acting is remarkably generic.

Relatedly, Aristotle is quite aware that things take on meanings or qualities only in reference to the things with which they are compared and that humans (as groups and individuals within) may assign wide ranges of labels and meanings to things. Like Mead and Blumer, Aristotle is attentive to matters such as knowing (vs. not knowing), acting with intention, attending to the future and past as well as the present, anticipating the viewpoints and reactions of others, and so forth.

Because Aristotle engages the study of the human condition in such comprehensive, focused, and detailed manners, it will not be possible to represent all that Aristotle has to say about deviance and the related matters of morality, regulation, involvement, and human relations, identity work, emotionality, and agency. Not only do these matters take readers into several of Aristotle’s texts, but Aristotle’s discussions of deviance and morality also are embedded in different ways within these texts.

Following a brief consideration of (1) Aristotle’s notions of causality, more sustained attention is given to (2) *Nicomachean Ethics* and (3) *Rhetoric*. Because Aristotle covers so much conceptual material in developing his statements, readers are cautioned about the necessarily compacted and partialized nature of the ensuing materials.

## Aristotle on Causality

Although Aristotle is often envisioned as an objectivist and this description is accurate with respect to Aristotle’s approach to the physical or material world, his work on formal logic (*Prior Analytics*), and his highly, analytic approaches (secular vs. theological; pluralistic vs. narrowly moralist; pragmatist vs. idealist) to the study of things more generally, Aristotle’s considerations of *human behavior* and community life are notably intersubjective and interactive in emphasis.

Relatedly, while Aristotle clearly respects the biological features of the human condition, as well as the material world in which people do things, Aristotle is acutely attentive to the ways in which people *enter into* the causal process as (minded) agents; in meaningful, speech enabled, learned, deliberative, interactive, and adjustive terms.

Unfortunately, this very central aspect of Aristotle’s approach to the study of the human condition has been extensively neglected or disregarded, particularly it seems by those who seek more simplistic religious or structuralist explanations of human behavior (and deviance).

Another reason that Aristotle has been viewed as an objectivist may revolves around what has become known as Aristotle’s “doctrine of the four causes” (as in composition, shape, direction, and mover). Although Aristotle clearly intended to encompass all physical instances in his statement on causation, he does *not* ignore human agency. Nevertheless, commentators on Aristotle often present these notions in highly truncated forms and have tended to focus, more simplistically, on physical or material notions of causality.

Operating at a highly abstract or generic level of knowing, Aristotle’s depiction of “the four causes” as stated in *Physics* (especially Book II: 194b-196a) and *Metaphysics* (Book I: 980a-983b; Book V: 1013a-1014a) focuses on (l) the matter or substance of which something is constituted (i.e., that of which it is made); (2) the shape or form that something assumes; (3) the emergent, directional (purposive in the case of human agents) features of the product or outcome; and (4) the mover of the process or source of the effect (including people as deliberative, interventional agents).

Those who examine either of the fuller texts (*Physics* or *Metaphysics*) will find, as well, that Aristotle not only recognizes that the number, variations, and interrelatedness of “causes” can be great indeed, but that he *also* envisions causes as terms that people invoke or assign to things in their quest to know things. Aristotle further observes that causality may be distinguished with respect to: potential, current, and past effects; natural and human causes; and accidental and intended human causes.^10^

Relatedly, when discussing human agency or the ways that people do things (see *Nicomachean Ethics* [1110a-1115a] or *Eudemian Ethics* [1222-1227a]). Aristotle is particularly attentive to people’s capacities to produce cause and effect in knowing and intentional manners. *Rhetoric*, *Poetics*, and *Politics* further attest to people’s capacities to shape or effect outcomes by influencing and resisting one another.

## Aristotle’s *Nicomachean Ethics*

[A]n act is compulsory when its origin is from without, being of such a nature that the agent, who is really passive, contributes nothing to it…

A voluntary act would seem to be an act of which the origin lies in the agent, who knows the particular circumstances in which he is acting. (Aristotle, Nicomachean Ethics, BIII, i [Rackham, trans.])

Although written to encourage more virtuous life-styles on the part of citizens and thus promote a more viable set of individual and community circumstances, Aristotle’s *Nicomachean Ethics* [*NE*] not only outlines Aristotle’s notions of virtue (and the failings thereof) but also represents a remarkably generic consideration of human reflectivity, deliberation, and interchange amidst a focused and more pervasive emphasis on biologically and linguistically enabled *activity*.^11^

Whereas Thomas Aquinas [1225-1274CE] 1964 represents a particularly notable exception, Aristotle’s *emphasis on activity* has been comparatively neglected by most commentators.^12^ This is most unfortunate since activity is central to understanding Aristotle’s conceptualization of the human condition.

Aristotle’s work on ethics or human choice and conduct (*Nicomachean Ethics*, *Eudemian Ethics*, *Magna Moralia)* represents only part of his analysis of human knowing and acting. Thus, in addition to Aristotle’s depictions of more scholarly practices of reasoning in *Categories*, *De Interpretatione*, *Prior Analytics*, *Topics*, *Sophistical Refutations*, *Physics,* and *Metaphysics*; readers should also be aware of Aristotle’s related, more generic considerations of mindedness in the human condition in *On the Soul*, *Sense and Sensibilia*, and *On Memory*; and Aristotle’s more direct discussions of human reflectivity, interchange, and relationships in *Poetics*, *Politics*, and *Rhetoric*.

Further, while developed as part of a larger agenda to develop a fuller comprehension of human affairs (*NE*, X: ix), Aristotle also envisioned *Nicomachean Ethics* as a foundational statement for political (from *polis* or city state) science or the analysis of the production and maintenance of social order in the community and a prelude to *Politics*.^13^

In the process of developing *Nicomachean Ethics,* Aristotle directly and consequentially deals with (1) *the human quest for good (goals, ends, objectives)*; (2) *virtue and vice* (as humanly engaged realms of activity); (3) *human agency* (with respect to voluntary behavior, deliberation and counsel, choice, practical wisdom, and activity); (4) *character* (as formulated, dispositional, and alterable); (5) *happiness* (with respect to pleasure, pain, virtues, and activity); (6) *emotion* (as experienced, developed), (7) *justice* (as in principles, law, and regulation); and (8) *interpersonal relations* (as in friendship, family, benefactors, and citizenry).

Because of the densely compacted nature of Aristotle’s discussions of these topics, it is not feasible to attempt to cover all of these matters. The following listing of chapter (conventionally referenced as books) divisions [with the names I have assigned to each chapter in brackets] may provide readers with an overall sense of this volume:

Book I [On Human Good]
Book II [Agency and Virtues]
Book III [Voluntariness, Virtues, and Vices]
Book IV [Virtues and Vices, continued]
Book V [Justice]
Book VI [Knowing, Deliberating, and Acting]
Book VII [Human Failings]
Book VIII [Friendship]
Book IX [Friendship, continued]
Book X [Pleasure, Activity, and Mindedness]

Whereas an attempt will be made to maintain the overall flow of *NE* while dealing with topics more pertinent to deviance within *NE*, it should be emphasized that much like the interactionists who have a more general theory of human group life, it is necessary to establish a broader, *pragmatist* base for Aristotle’s notions of deviance.

In what follows, I have extracted materials on Books I, II, III, V, VI, VII and X from a fuller interactionist consideration of Aristotle’s *Nicomachean Ethics* that can be found in Prus (2007a). Readers are encouraged to examine the more extended synoptical statement available in *Qualitative Sociology Review* (Prus 2007a) as well as the much fuller statement available in Aristotle’s *Nicomachean Ethics*.

### Book I [On Human Good]

Aristotle begins *NE* (I: i) by observing that the *good* is that (goal, end, purpose) to which particular and/or general sets of human activities are directed. In developing this position, Aristotle notes that the various arts and sciences are directed toward different objectives. He also says that some pursuits may be subsumed by others and that these broader ends appear more worthwhile than the lesser pursuits (and objectives) that they encompass.

Aristotle (*NE* I: ii) extends these notions further, arguing that the supreme good would be that which is most consequential for the conduct of human life. Focusing on the human community (*polis*) for which (and in which) all human arts and sciences are developed, Aristotle contends that the ultimate good should be approached within the context of a *political science*. Emphasizing the centrality of the community over the individual, Aristotle defines the good of the people (in the community) as the primary objective of the science of politics. Still, Aristotle (*NE* I: iii) cautions readers that one should not expect similar levels of precision across all areas of human study and to recognize the tentative nature of his present statement.

Whereas Aristotle (*NE* I: v) identifies four pursuits that people commonly associate with happiness – sensate pleasures, political fame, study, and wealth, he also alerts readers to the problematic qualities of people’s quests for happiness.

After noting that it is people’s minds and capacities for virtuous or noble activity that importantly distinguishes humans from other animals (*NE* I: vi), Aristotle observes (*NE* I: ix) that people’s conceptions of happiness can be highly diverse. Relatedly, although the more virtuous notions of happiness are best achieved through study and effort, he says that people who work to accomplish things tend to be happier with their results than those who gain similar ends through gifts or fortune. Accordingly, the goal for a political science is to promote more virtuous standpoints on the part of people and to encourage their participation in noble realms of activity. In discussing these objectives in the materials following, he (*NE* I: xi) will be mindful of the nonrational bodily quality of humans as well as people’s minded capacities for reasoning.

### Book II [Agency and Virtues]

Aristotle (*NE*, II: i) begins his consideration of moral virtues by distinguishing these virtues of habits from intellectual virtues.^14^ Whereas intellectual excellences (discussed later, *NE*, VI) are seen as contingent on explicitly developed instruction and experience, *moral virtues* derive from people’s earlier habits (i.e., tendencies, dispositions, or more persistent ways of doing things). Although Aristotle sees people as born with capacities for both intellectual and moral development, he explicitly states that none of people’s moral virtues are inborn or determined by matters (as in “stimuli”) that are external to people.

While Aristotle (later, *NE*, II: iii) defines moral virtues and vices as contingent on people acting appropriately (or inappropriately) with respect to pleasure and pain, he envisions moral virtues and vices in developmentally acquired terms. Accordingly, for Aristotle, people’s habits have a notably different quality from people’s more informed, knowledge-based, deliberations (as in wisdom, thought and associated judgments).

Thus, Aristotle (*NE*, II: i) states that people’s moral virtues are the products of people’s earlier practices. They reflect the habits that people develop around *ways of doing things* and the types of associations that particular people develop with others. Because people’s habits begin to develop early in life, Aristotle contends that people’s childhood training can be particularly consequential for shaping one’s character and dispositions in this regard.^15^

Continuing, Aristotle (*NE*, II: ii) notes that one of the problems pertaining to people’s conduct is that people, *as agents*, must decide what is most appropriate to do in the circumstances at hand. Recognizing the highly variable nature of human conduct, Aristotle says that conceptualizations or models dealing with this subject matter will necessarily be somewhat imprecise.

Aristotle (*NE*, II: iii) also states that considerations of moral excellences are to be understood centrally with respect to people’s concerns with joy or pleasure and sadness or pain. However, while people pursue things because of the attractions or pleasures they afford and avoid things because of the sorrows or punishments they associate with particular things, Aristotle notes that people’s notions of pleasure and pain need not correspond with things that others would so define.^16^

Still, Aristotle defines moral virtue as a matter of acting in the best or most honorable way with respect to people’s senses of joy and sorrow. Conversely, vice is defined as the failure to act in appropriate fashions with regard to pleasure and pain.

Aristotle then isolates *three motives of choice* that help define *acts* (not synonymous with dispositions) as morally virtuous: noble vs. common (or base) interests; advantageous vs. harmful considerations; and pleasure vs. sadness orientations.

Relatedly, in order *for acts to be considered morally virtuous*, Aristotle (*NE*, II: iv) says that certain criteria must be met. Thus, people must (a) act with knowledge about what is being done; (b) act with intention; and (c) act from an existing habitual tendency or disposition rather than from an intellectual or reasoned standpoint.^17^

Aristotle (*NE*, II: v) subsequently distinguishes virtues from people’s emotions and capacities to act. While virtues may involve emotions such as anger or shame, and are contingent on people’s capacities to act, Aristotle says that moral virtues most basically represent habits or dispositions to act. In discussing the moral virtues – as desirous standpoint categories of acting and two associated sets of opposite extremes (failings or vices), Aristotle (*NE*, II: xv) delineates a set of moral virtues along the lines following:

- Brashness – *Courage –* Cowardice
- Extravagance – *Personal Liberality* – Stinginess
- Crass Display – *Public Generosity* – Miserliness
- Vanity – *Honor* – Disregard
- Ambitiousness – *Dedication* – Inattentiveness
- Irritableness – *Gentleness* – Spiritlessness
- Boastful – *Sincerity* (regarding self) – Self Depreciating
- Buffoonery – *Congeniality* – Distancing
- Pretentiousness – *Friendliness –* Rudeness
- Shameless – *Modest –* Shyness
- Envious – *Fair –* Malicious
  As a general “rule of thumb” regarding the moral virtues, Aristotle encourages people to adopt midpoints in both their conceptions of self and the ways they relate to others. People’s tendencies, emotionalities, and preferences toward either extreme are seen in more vice-like terms.
  Observing that it can be difficult to achieve the midpoints in actual practice, Aristotle (*NE*, II: ix) encourages people to strive for more general, virtuous standpoints in their activities. Even so, he adds, people’s conceptions of midpoints and variations thereof will be matters of (relative) human judgment.

### Book III [Voluntariness, Virtues, and Vices]

Aristotle assumes two tasks in Book III. The first and most important matter for our purposes, is his consideration of human *responsibility*. His second objective is to begin a more detailed examination of the specific moral virtues.

Stating that virtue revolves around emotions and actions, Aristotle (*NE*, III: i) says that *praise and blame* are appropriate only when people engage in *voluntary actions*. To this end, Aristotle embarks on considerations of voluntary and involuntary actions and the related matters pertaining to choice, deliberation, ignorance, and opinion, as well as an identification of several of the components of action.

Noting that the issue of *actor responsibility* is apt to be of concern to people assigning rewards and punishment to others as well as to students of human conduct, Aristotle says that actions are generally characterized as *involuntary* when people are able to exercise little control over the direction of their action either as a result of compulsion or ignorance.

Aristotle also recognizes that many instances of action are *mixed* in effect, whereby people may have some abilities to choose or control things in the setting, but may still encounter other kinds of limitations. As well, Aristotle distinguishes cases of more general ignorance (wherein one does not know many things) from those instances in which people lack a more specific awareness of some feature or circumstance of the act at hand.

Accordingly, Aristotle distinguishes a number of *features of the situation* that people may consider in assigning voluntary or involuntary status to those involved in particular episodes. There are (a) the agent; (b) the act; (c) the thing (i.e., person or other objects) affected by the act; (d) the instruments or devices employed in conducting the activity; (e) the outcomes of the act; and (f) the manners (e.g., gently or violently) in which particular acts were performed. Relatedly, Aristotle observes, while people (as agents) often know about these things in advance, when people are unaware of certain features of acts or make mistakes regarding any of these components, this may be seen to introduce an involuntary feature into the event at hand.

*Voluntary acts* Aristotle notes, refer to situations in which (a) some activity is initiated by the person and (b) the person is more completely aware of all of the aspects of the situation pertaining to that activity. Aristotle adds that it should not be presumed that acts that are generated amidst anger or desire are involuntary. In part, he explains, if people can voluntarily act in noble terms under these conditions, it makes little sense to characterize ignoble acts based on the same explanatory motives as involuntary.

Aristotle (*NE*, III: ii) next turns to the matter of *choice*. Because people may not be able to act as they desire or intend, Aristotle reasons, people’s choices may provide better understandings of their virtues than their eventual actions. Aristotle views choice as a voluntary act, but notes that not all voluntary acts entail (deliberative) choice.

Although people often describe choice as desire, passion, wish, or opinion, Aristotle says that these viewpoints are mistaken. Choice is not a desire or other standpoint on things. Choice involves a selection between two or more items and implies some *deliberative activity*. Likewise, while people may have definite viewpoints, opinions, or preferences pertaining to things, it is not to be assumed that people will automatically make choices that correspond to those ideas.

Aristotle (*NE*, III: iii) then addresses the topic of *deliberation* in more direct terms. Rather than deliberate about everything, Aristotle says, people tend to deliberate about things over which they have some control and seem attainable through their activities. As well, he adds, people deliberate about things about which they are more uncertain. And, when they consider particular issues important, people are more likely to involve others or seek counsel in their deliberations.

Continuing, Aristotle notes that deliberation constitutes a form of investigation wherein people may consider, in varying degrees of detail, all aspects of the situation about which choices are to be made. As well, because all actions are purposive or intended to do or accomplish something, deliberation revolves around the ways that one might attain things.

Aristotle (*NE*, III: iv) reminds readers that because wishes are for certain outcomes or ends, people’s wishes or desires are to be distinguished from choices and deliberation about how to achieve particular wishes or other ends.

Aristotle (*NE*, III: v) then turns more directly to *virtues* and *vices*. Having excluded certain actions from praise and blame because they are involuntary in some way, Aristotle argues that *both* virtues and vices are to be understood as *voluntary matters*. Still, Aristotle reminds readers, people are not able to control their own dispositions as readily as many other features of their actions.^18^ [Note: despite their notably illustrative relevance for a variety of people’s qualities of “character” and people’s implied moral evaluations of others and themselves along these lines – and their subsequent implications for the potential reshaping of people’s characters I have omitted Aristotle’s depictions of courage and self-regulation in Book III and his discussion of the other moral virtues in Book IV.]

### Book V [Justice]

Although a discussion of justice represents a break of sorts from Aristotle’s consideration of deviance (involvements) *as activity*, it points to another dimension of Aristotle’s attentiveness to community (and political) life as well as an appreciation of people’s concerns with the regulation of deviance (and obtaining compensation for losses associated with acts perpetrated by others). Thus, while continuing his broader discussion of the moral virtues, Aristotle focuses Book V of *NE* more directly and consequentially on *justice* as a socially engaged feature (virtue) of community life.

After noting that people use the terms “just” and “unjust” in several ways, Aristotle (*NE*, V: i) introduces two themes that will become central to his analysis. These pertain to people (a) being law abiding and (2) receiving fair or equitable shares of things.

Aristotle states that what is lawful is a matter of legislation, noting that what this actually includes and how this is decided reflects the type of government in effect at the time. Thus, Aristotle defines justice in reference to the political body in charge of the community.

He also argues that justice should be envisioned as the most consequential of the moral virtues because justice is engaged mindfully of others. Justice, thus, is seen to represent a community standpoint that goes beyond the interests of particular individuals. While virtue is envisioned as an individual disposition to act in an ennobling fashion, justice may be seen to epitomize virtue because it is directed toward the good of the community in a more comprehensive sense.

Continuing, Aristotle (*NE*, V: ii) reaffirms the centrality of justice as a virtue and injustice as a vice. Aristotle then distinguishes distributive or proportionate justice from remedial or corrective justice.

Aristotle defines *distributive justice* as an equitable, proportional distribution among people who are working with pre-established notions of comparative merit. Thus, for instance, citizens or equal partners may share things equally among themselves but are not obliged to share things (at least not in the same proportions) with those who do not possess this status.

*Remedial* or restitutive justice is intended to correct imbalances that are attributable to the undesired effects of people’s behaviors on particular others. Thus, the negatively affected parties may seek restitution for their losses or pursue other kinds of remedial services for themselves or correctional treatments for the perpetrators. Remedial justice may involve situations in which the aggrieved parties had participated voluntarily (as in marketplace transactions) in situations with the alleged perpetrators, but the injured parties also may have had things involuntarily imposed on them (as in theft, robbery).

Focusing more directly on restitutive justice, Aristotle (*NE*, V: iv) states that people go to judges to seek justice because judges represent the personification of justice, adding that in some locales judges are labeled *mediators* because people presume that judges will invoke a midpoint (or median) in determining what is just to the parties involved.

In discussing the problem of determining justice (as in defining damages and repayments), Aristotle (*NE*, V: v) explicitly acknowledges *money* as a particularly valuable standard. While observing that the value that people put on money will fluctuate somewhat (as with other things), Aristotle notes that money not only facilitates exchange of all sorts but money also represents a resource that people conveniently may use at future points in time.

Aristotle (*NE*, V: vi) then discusses *political justice*, applying this term to people who are free and equal with respect to one another within a particular community context. Relatedly, Aristotle notes, this is why people emphasize the law over a ruler. He says that the appropriate function of the ruler is to be guardian of justice.

Subsequently, Aristotle (*NE*, V: vii) distinguishes two conceptions of political justice. One is *natural justice*, wherein the same notions of justice would apply to everyone, everywhere. The other, Aristotle describes as *conventional justice* and envisions it as having a local quality. Aristotle insists that there is a natural justice, while observing that all rules of justice (presumably as invoked) are variable.

In a similar manner, Aristotle points to a distinction between things considered just or unjust and actual conduct that is just or unjust.

Aristotle (*NE*, V: viii) then notes that *considerations of just and unjust conduct* are contingent on people (a) acting in voluntary manners, (b) exercising choices, and (c) acting in ways that are mindful of the outcomes that could be expected under the circumstances.

Thus, Aristotle observes that the penalties associated with injury may be minimized when injurious acts are done without evil intent, are due to outside influences or constraints, or reflect uncontrollable instances of passion.

Subsequently, Aristotle (*NE*, V: ix) states that things prescribed by the law are actions but that actions need to be qualified when matters of justice are invoked. Thus, while people may contemplate acting in certain ways, Aristotle notes, it is not easy to know exactly how to act so that the result would be considered a just or appropriate act.

Next, Aristotle (*NE*, V: x) briefly comments on the relationship of equity and justice, noting that the two are not synonymous. Aristotle suggests that concerns with equity, as a concern with fairness to the parties at hand, may provide a corrective of sorts to justice that has a more abstract or generalized application. Aristotle also notes that because laws are intended as general statements, they cannot be expected to fit all cases.

### Book VI [Knowing, Deliberating, and Acting]

Having discussed the moral virtues (Books III and IV) and people’s conceptions of justice in Book V, Aristotle subsequently focuses on the intellectual virtues in Book VI. He begins by saying that it is not enough just to give instruction on conceptions of virtues. Subsequently, Aristotle (*NE*, VI: ii) identifies three aspects of the human *psyche* that control action and people’s definitions of the truth. These are sensation, desire, and thought.

After stating that sensations cannot in themselves generate rational (as in minded or deliberative) action, Aristotle observes that desires (as in moral virtues) provide direction, but that people’s desires also are inadequate for explaining human behavior.

Thus, Aristotle states, the more effective cause of human action is *thought* in the form of choice. Still, he observes that, thought in itself moves nothing. Thought is consequential in causal terms *only when* it is directed toward some ends and when it is manifested in action. Aristotle continues, stating that people, thus, are *originators of action*, by unifying desire and thought.

Aristotle (*NE*, VI: iii-viii) discusses five ways that people may assess their knowledge of things. First, there is scientific knowledge as a reference point – denoting inquiry into instances and comparative analyses as well as the related matters of instruction and learning. Second, people can acquire more viable knowledge of things as a matter of art or technique wherein they rely on focused, sustained realms of practice and minded, reasoned adjustments.

Third, confidence in knowing also may be gained through phronesis or prudence – recognizing human capacities to more fully deliberate about things and make more carefully reasoned definitions of the matters at hand.

Fourth, a more reliable sense of knowing can be achieved through the acquisition of wisdom – wherein attains a more comprehensive experiential knowledge base than that implied in scientific knowledge per se – one develops a broader more extended stock of knowledge for assessing situations.

Aristotle (*NE*, VI: ix) then reengages phronesis or the matter of careful reasoning further – saying that when more extended deliberation is combined with a fuller sense of wisdom about things, it is under these circumstances that people are likely to know things in ways that allow them to make the best decisions. Because deliberation deals with uncertainties, it implies a process of investigation. However, in further contrast to science investigation (that deals in concepts of a more universal sort), the emphasis in deliberation revolves around the understanding and anticipation of specific instances or applications.

Aristotle adds that whereas superior intelligence may enable people to make quicker judgments as well as develop more detailed understandings of things, informed, reasoned thought is much more important than intelligence for viable decisions and moral conduct.

### Book VII [Human Failings]

While focusing on some of the problematic features of the human will (and taking issue with Socrates and some other theorists), Aristotle notes that even though notions of pain and pleasure are particularly relevant to the moral virtues that people develop, it is important to recognize the variety of viewpoints people may adopt in defining pain and pleasure. He observes that people may derive pleasure from opposite states and one should not assume that certain things are automatically pleasurable.

In particular, Aristotle stresses the point that pleasure is an *activity* and, as such, is more encompassing and different from a (non-minded) process. Likewise, given the complexity of the human mind he observes that variations can be important in the ways that people experience (and redefine aspects of) pleasure.

### Book VIII [Friendship] Book IX [Friendship]

While Aristotle’s analytically detailed consideration of friendship in Books VIII-IX has important implications for people’s involvements in deviance as well as more personalized realms of virtue, it is not be possible to deal with these matters in the present paper (see Prus 2007a: 29-33).

### Book X [Pleasure, Activity, and Mindedness]

The material from Book X also is less central to the immediate discussion of deviance but some of it is included because it helps portray Aristotle’s views of pleasure as a minded process (vs. pleasure as a motivational force that is often presumed to prompt deviance).

After reviewing and commenting on some other philosophers’ notions of pleasure, Aristotle (*NE*, X: iv) intends to establish his own views on pleasure.

Aristotle begins by claiming that pleasure is not a specific thing but has a more unified or encompassing quality. Pleasure, thus, cannot be envisioned as a physical motion or a process in itself or even the result of a process. Likewise, while Aristotle contends that the potential for pleasure is greatest when people’s capacities for sensory perception are at their functional best, Aristotle wants to emphasize that it is the *mind* (not one’s physiology per se) that is stimulated. It is through the mind that people experience pleasure.

However, pleasure is *not* simply a matter of (minded) definition in this respect, nor is pleasure contingent exclusively on motions (behaviors) or sensations that human bodies encounter. Instead, Aristotle contends, people’s experiences of pleasure necessarily reflect *the interlinkages of action, sensations, and minded focusing*. Thus, for Aristotle, pleasure is a minded, embodied, and processually developed activity.

### *Nicomachean Ethics* in Perspective

Aristotle’s *Nicomachean Ethics* is important for the study of deviance not only because Aristotle approaches wrongdoing or vice as a natural aspect of human group life but he also stresses the centrality of activity, particularly of a meaningful, deliberative, and moral (directional) sort for understanding all instances of behavior.

For Aristotle, matters of voluntariness, intentionality, deliberation, and associated aspects of human agency are central to all considerations of group life and people’s behaviors and relationships within. This holds for noble and more routine activities as well as those considered most disreputable. Similar matters also apply to people’s notions of law and justice as well as people’s attempts to provide correctives to undesired human practices.

Given (a) the overall affinities of Aristotle’s conceptualizations of human knowing and acting with the viewpoints developed within symbolic interaction and (b) the many junctures he provides for subsequent thought, analysis and research, Aristotle’s contributions to an understanding of deviance as a humanly engaged process in *Nicomachean Ethics* remain remarkable by contemporary standards. Indeed, there is much to be appreciated in Aristotle’s notions of purposive behavior, reflectivity, habits, deliberation, choice, action, culpability, and justice as these pertain to human knowing and acting.

Beyond the instructive comparative resources that one finds in *NE*, this text also provides a great many analytic insights for contemporary scholars to consider with respect to human knowing, acting, and interchange. Still, while building on this exceptionally potent foundational base, Aristotle has yet more to offer to students of deviance in *Rhetoric*.

Thus, whereas the broader explanation of human behavior that Aristotle generates in *Nicomachean Ethics* will better enable readers to appreciate the analytical standpoints Aristotle develops in *Rhetoric*, Aristotle’s *Rhetoric* deals much more directly with contested realms of identities, activities, and events than does *Nicomachean Ethics*.

## Aristotle’s Rhetoric

But since the object of Rhetoric is judgment–for judgements are pronounced in deliberative rhetoric and judicial proceedings are a judgment–it is not only necessary to consider how to make the speech itself demonstrative and convincing, but also that the speaker should show himself to be of a certain character and should know how to put the judge into a certain frame of mind. For it makes a great difference with regards to producing conviction–especially in demonstrative, and, next to this, in forensic oratory–that the speaker should show himself to be possessed of certain qualities and that his hearers should think that he is disposed in a certain way towards them; and further, that they themselves should be disposed in certain way towards him. (Rhetoric, BII, i [Freese, trans.])

Although much of the material dealing with Aristotle’s *Rhetoric* has been extracted from Prus (2008a), readers are encouraged to examine the more extended synoptical statement available in *Qualitative Sociology Review* (Prus 2008a) as well as Aristotle’s yet much fuller text, *Rhetoric*.

In developing *Rhetoric*,^19^ Aristotle provides a remarkable philosophic analysis of *rationality in the making*. He presents readers with a comprehensive, highly instructive depiction of *persuasive interchange* as a strategically engaged, linguistically accomplished (and potentially contested) process.

Thus, while Aristotle discusses (1) the characters (reputations), abilities and tactical ploys of *speakers*, and (2) the contents of people’s *speeches* and the ways in which speakers present their cases to judges, Aristotle even more centrally (3) focuses on the ways that speakers may appeal to (and alter) the viewpoints of the *judges* to whom messages are pitched.^20^

Whereas Aristotle’s notions of deviance are articulated within a broader agenda of enabling speakers to assume more competent or persuasive roles as rhetoricians, Aristotle very much intends that those whom he instructs will have a full and detailed knowledge of that which they address. It is in this context that Aristotle develops materials within *Rhetoric* that deal with matters of human conduct raised in *Nicomachean Ethics* more generally and with wrongdoing and judicial applications in greater detail.^21^

In his typical, highly analytic manner, Aristotle provides readers with much more material than can be introduced in a statement of the present sort. Thus, while maintaining the overall flow of Aristotle’s *Rhetoric*, the material presented here is to be recognized as partialized in its presentation.

The headings used here (my own wordings) acknowledge the range of materials that Aristotle develops in this text but they still convey only a highly limited sense of Aristotle’s *Rhetoric*. More specific “Book and chapter” references will be provided for the materials considered in the ensuing discussion.

Book I

Defining Rhetoric

Realms and Emphases of Persuasion

 Deliberative (political) Rhetoric

 Epideictic (evaluative) Rhetoric

 Forensic (judicial) Rhetoric

Forensic Rhetoric

 On Wrongdoing

 On Justice

 On Judicial Contingencies

Book II

Pursuing Favorable Decisions

Maximizing Credibility

Attending to Emotionality

 Anger and Calm

 Friendship and Enmity

 Fear and Confidence

 Shame and Shamelessness

 Kindness and lnconsideration

 Pity and Indignation

 Envy and Emulation

 Acknowledging Generalized Viewpoints

Enacted Features of lnfluence Work

 Generating and Refuting Arguments

 Possibilities and Probabilities

 Arguments, Examples, and Enthymemes

 Contesting Cases

Book III

Amplifying and Diminishing Images

Arranging and Deploying the Components

 Proem or Introduction

 Narration or Account

 Proofs or Claims and Counterclaims

 Peroration or Concluding Statements

In what follows, I (a) briefly consider rhetoric as a broader field of persuasive endeavor and then focus more directly on (b) forensic (also legalistic, judicial) rhetoric – giving attention to wrongdoing, justice, and judicial contingencies, before attending to the matters of (c) pursuing favorable decisions – as this pertains to credibility and emotionality and (d) the enacted features of persuasive interchange.

While placing supreme emphasis on human knowing (and acting) as a form of excellence, Aristotle also recognizes that, regardless of whether people’s representations of things are accurate or otherwise, *rhetoric* (as persuasive communication) *becomes the route to a great many instances of human knowing, decision-making, and acting*. More than a technique or procedure, thus, rhetoric is a medium or communicative process through which people share meanings of things with others in a most fundamental sense.

As well, since people may embark on influence work in any variety of settings, rhetoric is applicable to court-related proceedings, community celebrations, management practices, internal community policies and decisions, and intergroup (interstate, international) relations as well as interpersonal relations. It is because of this exceedingly broad base that the study of rhetoric is so important for comprehending community life.

Recognizing that most readers are apt not to be familiar with Aristotle’s *Rhetoric*, the overall flow of this volume has been maintained. This should enable readers to establish more direct links with Aristotle’s statement and, hopefully, encourage use of this material for their own studies of human relations. At the same time, though, readers are cautioned that, far from amplifying Aristotle’s analysis, this statement only partially captures the depth, detail, and potency of Aristotle’s *Rhetoric*.

## Defining Rhetoric

Establishing an orientational frame for embarking on influence work, Aristotle (BI, i-ii) states that *rhetoric represents the study of the available means of persuasion on any subject matter*. He also observes that his concern is not limited to matters of successful techniques but represents an attempt to discover the ways in which persuasion work may be engaged in the instances in which this takes place.

Largely disregarding Plato’s intense condemnations of rhetoric, Aristotle notes that rhetoric (like other arts or technologies) may be used for variety of ends. Whereas rhetoric relies primarily on linguistic communication, Aristotle’s *Rhetoric* clearly attests to the *limitations of words* as persuasive elements in themselves. Thus, throughout this volume, Aristotle is highly attentive to (1) the *speaker* (interests, abilities, and images of the speaker), (2) the *speech* (contents, ordering, and presentation), and (3) the *audience* (dispositions, viewpoints, inferential tendencies, and resistances). He also is mindful of (4) the anticipatory, adjustive interchanges that oppositionary speakers may develop as they vie for the commitments of the auditors in the setting.

Aristotle divides rhetoric into three major categories (BI, iii-iv), relative to speakers’ primary objectives. These are (1) deliberative, (2) forensic, and (3) epideictic rhetoric.

*Deliberative* or political rhetoric is intended to encourage people to act or, conversely, to discourage them from acting in certain ways. Concerned with decision and commitment making processes, deliberative speaking presumes a distinctively futuristic orientation. While not minimizing its importance, Aristotle acknowledges the nature of people’s community-based concerns, forms of government, and the more generic lines of action that might represent points of interchange in this highly compacted statement on deliberative rhetoric.

*Forensic* or judicial rhetoric (discussed in extended detail later) is used to charge others with offenses of some sort or, relatedly, to defend people from the charges of others. Whether these claims are invoked on behalf of individuals, groups, or the state, forensic speeches deal primarily with matters alleged to have happened in the past.

Referring to the praise or censure of people or things, *epideictic* or demonstrative rhetoric has a more distinctively evaluative purpose. It largely deals with celebrations or condemnations of some target or humanly-experienced circumstances. These instances of evaluative rhetoric typically are developed around some present (as in recent or current) person or group, occasion, event, or situation.

Still, mindful of the notably complex and sophisticated legal system in effect at Athens,^22^ most of Aristotle’s *Rhetoric* deals with judicial or forensic rhetoric. Although the term deviance as used by interactionists extends beyond things that may involve criminal or civil court proceedings, it is difficult not to appreciate the vast array of related conceptual insights that Aristotle introduces and pursues in his consideration of judicial cases.

## Forensic Rhetoric

Attending to the comparatively extended and sophisticated legal system in effect at Athens, most of Aristotle’s *Rhetoric* deals with judicial or forensic rhetoric. Although the term deviance as used by interactionists extends beyond things that may involve criminal or civil court proceedings, it is difficult not to appreciate the vast array of related conceptual insights that Aristotle introduces and pursues in his consideration of judicial cases.

Focusing on matters of accusation and defense, Aristotle’s consideration of forensic rhetoric is conceptually dense, sophisticated, and highly instructive. Thus, even as he frames the analysis at a more preliminary level, Aristotle provides readers with compelling insights into (1) wrongdoing, (2) justice, and (3) judicial contingencies. Given our emphasis on deviance, these topics are given somewhat greater attention.

### On Wrongdoing

While acknowledging people’s inadvertent and unwitting *involvements in some instances of wrongdoing*, Aristotle approaches people’s involvements in wrongdoing or deviance in ways that *directly parallel* his views on the ways that people engage in other [nondeviant] activities – *as meaningful, deliberative, goal-oriented pursuits*.

In what clearly anticipates the position developed by twentieth century pragmatists (e.g., Mead 1934) and interactionists (Becker 1963; Blumer 1969), Aristotle does not require separate theories for the deviants and nondeviants, but rather presents one theory that enables scholars to examine all instances of meaningfully developed human behavior.

Attending to both written legislation *and* unwritten laws (or generalized understandings) in forensic arenas, Aristotle not only outlines (a) people’s *motives for wrongdoing*, and (b) the various *states of mind* that people might adopt in pursuing these activities, but he also considers (c) those who are *targets* of these endeavors and the ways in which targets (e.g., as victims, precipitators) enter into the activities in question.

Addressing human action in judicial settings, Aristotle (BI, X) briefly delineates seven bases or causes of human behavior, including chance, compulsion, nature, custom, will, anger, and appetite (pursuit of pleasure).

Aristotle does not sort these motivational themes out in much detail but instead focuses on the *voluntary*, *deliberative* activities associated with the *pursuit of pleasure* or desired experiential states more generically.

Then, using pleasure as a centralizing concept with which to comprehend the known, meaningful features of action, Aristotle (BI, X-XI) proceeds to illustrate how all of the voluntary aspects of the preceding set of causes involve the pursuit of pleasure (notions of happiness and the avoidance of discomfiture).

Aristotle is attentive to people’s capacities to experience bodily sensations, but it is inaccurate to envision Aristotle as a physiological hedonist or psychological reductionist. Pleasure and pain, thus, are defined not as stimuli but in terms of people’s *desired end-states*.

These could include people’s quests for more direct physical sensations, but also would encompass the values people place on the development of the intellect, moral pursuits, or concerns about the well-being of others, for instance.

Beyond speakers ascertaining and pitching to audiences in terms of *things that these particular auditors value*, Aristotle deems it essential that speakers *understand* the motivational and engaged features of *human agency*.

In addition to establishing in the relevance of *memory* (recollection) and *hope* (anticipation) for people’s conceptions and pursuits of pleasures (and pains), Aristotle also discusses the *role of others* in these endeavors.

Hence, people’s notions of and quests for, pleasure involve their participation with others in such things as friendships, persuasive endeavors, and instances of rivalry, amusement, learning, admiration, and beneficiary roles, as well as attending to others as reference or comparison points.

Having established an operational base, thus, Aristotle (BI, XII) asks when people are apt to engage in wrongdoing. Assuming that people desire certain objectives and envision ways of achieving these ends, Aristotle states that people are more likely to actively assume agent or *perpetrator roles* when they think they (a) can accomplish the acts in question, (b) will escape detection, and (c) if detected, would avoid punishment, or (d) if they expect to experience punishment, anticipate that the gains would offset the losses.

Among those whom Aristotle identifies as inclined to *assume higher levels of impunity* in reference to their own acts are people who (a) are more talented in circumventing culpability more generally; (b) envision themselves to have more friends and supporters; (c) anticipate greater influence with injured parties or judges; and (d) seem like inappropriate (unfitting) candidates for the activities in question by others by virtue of their personal qualities or situations.

As well, Aristotle also envisions people as more likely to presume immunity from penalty when they (e) have convenient ways of concealing activities or easy ways of disposing of things; (f) have the means of influencing judges or otherwise averting penalties; (g) feel they have nothing to lose; and (h) perceive the gains to be close at hand or greater, while losses seem distant or less consequential. As well, Aristotle notes, those who (i) think that certain activities would generate prestige among certain of their associates also seem likely to act with a greater sense of impunity.

After discussing both (1) the attractions that people may develop for various wrongdoings and (2) people’s tendencies to assume roles as perpetrators, Aristotle (BI, XII) proceeds to (3) a consideration of *the targets* of these activities.

Acknowledging a wide range of targets, from friends (as easy, more trusting) and enemies (as more enjoyable), to those who are nearby (offering more immediate advantage) or distant (less prepared to resist), Aristotle observes that some people may be easier targets as a consequence of their tendencies to avoid pursuing offenders. This includes those who: do not want to be bothered with such matters; wish to maintain current levels of dignity; have been harmed many times before; are held in disgrace; are visitors to, or temporary residents in, an area; and, themselves, are guilty of similar or related offenses.

Aristotle also notes that people may define others as more viable targets for negative behaviors when they: anticipate undesirable treatment from those targets; expect that they can compensate targets for their losses; or envision others as acting negatively toward those targets.

### On Justice

As with *Nicomachean Ethics* (Book V), Aristotle engages the topic of justice in *Rhetoric*. Here, however, he is more focused on justice as an *enacted* feature of community life. Quite directly, then, Aristotle (*Rhetoric*, BI, XIII) provides still more insight in the deviance-making process through his considerations of *written law*, *natural law*, and *equity*.

Continuing his elaboration of just and unjust actions (and judicial cases more specifically), Aristotle (BI, XIII) distinguishes (1) the particular laws developed by communities of people from (2) a universal (presumably divinely-inspired or naturally emergent) law that is taken to transcend particular or local notions of justice, and (3) the specific conceptions of equity (and inequity) that speakers or others may invoke.

Even though the prosecutions he discusses are based primarily on (a) written laws, he observes that speakers may invoke notions of (b) natural law and (c) equity (introduce “fairness” as a reference point) along with (d) other aspects of written law in pursuing and contesting the cases at hand.

Next, Aristotle (1) delineates injustices perpetrated against communities from those conducted against individuals, (2) qualifies people’s activities in reference to degrees of intentionality; and (3) observes that perpetrators commonly define their acts in terms that are at variance from the definitions promoted by complainants.

Aristotle subsequently addresses *equity* as a concept of justice that speakers may use to challenge the formalities or technicalities of written law. When emphasizing equality or fairness, speakers endeavor to shift emphasis from (a) the legalistic concerns with the letter of the law and (b) the particular activities in question, to considerations of (c) the intent of the law, (d) the motivational principles of the agent, and (e) the willingness of the involved parties to pursue equitable arrangements through arbitration.

The next issue Aristotle (BI, XIV) addresses with respect to justice is the degree of *indignation*, blame or condemnation that audiences associate with people’s instances of wrongdoing.

Among the acts apt to thought *more blameworthy* are those that (a) violate basic principles of the community; (b) are defined as more harmful, especially if more flagrant and offer no means of restoration; (c) result in further (subsequent) injury or loss to victims; (d) are the first of their kind; (e) are more brutal; (f) reflect greater intent to harm others; (g) are shameful in other ways; and (h) are in violation of written laws. Thus, Aristotle lists a series of contingencies that he thinks are likely to result in someone’s activities being seen as more *reprehensible* by judges.^23^

#### On Judicial Contingencies

Aristotle (BI, XV) also addresses a realm of argumentation that is peculiar to *judicial oratory*. These revolve around (a) formalized laws, (b) witnesses, (c) contracts, (d) torture, and (e) oaths.

Returning to his earlier distinctions between written law, universal law, and equity, Aristotle indicates how speakers whose cases are at variance with the *written law* may appeal to notions of universal law and equity, while those whose cases are supported by written law may insist on the primacy of moral integrity and wisdom of the written law.

When dealing with *witnesses*, Aristotle acknowledges the wide variety of sources (including ancient poets and notable figures; contemporary characters, and proverbs) that speakers may use to provide testimonies for or against cases.

While noting that resourceful speakers have an endless set of witnesses on which to draw, Aristotle is also attentive to those witnesses who claim to have direct knowledge of the specific events at hand.

Relatedly, where speakers can provide direct witnesses to events, they may strive to enhance witness credibility, whereas speakers who do not have such witnesses would normally try to discredit the former and argue for the importance of the judge’s independent wisdom. Aristotle urges speakers to adopt somewhat parallel enhancing and denigrating tactics when dealing with *contracts* involving courtroom adversaries, evidence gained through *torture*, and the use and avoidance of *oaths*.

## Pursuing Favorable Decisions

Envisioning the preceding elements as more unique to forensic rhetoric, Aristotle (BII, I) turns to what he describes as the *art of rhetoric*. While not disregarding the context or the apparent matters of issue in particular instances, the focus is on presenting cases (on one side or the other) in strategically more effective manners.

Here, Aristotle focuses on the matters of developing emotional appeals, constructing cases, and presenting materials to judges. The emphasis, as well, shifts more directly to the task of *securing favorable decisions* in deliberative occasions *and* judicial cases.

Thus, before focusing on the more overtly enacted features of rhetoric, Aristotle addresses (1) the foundations of credibility, (2) people’s experiences with an assortment of emotions pertinent to influence work; and (3) the generalized viewpoints of particular categories of people.

### Maximizing Credibility

Aristotle’s statement on credibility asks when speakers’ claims are apt to be considered viable by judges. Succinctly outlining a theory of trust or credibility, Aristotle (BII, I) posits that audiences are likely to place greater faith or confidence in those speakers (as characters) who are thought to (1) display good sense in judgment, (2) possess excellence of capacity (competence, honor), and (3) act in ways consistent with the audience’s (advantageous) viewpoint in mind. The implication is that those who achieve credibility on the part of others will be heavily advantaged in their subsequent communications with others.

### Attending to Emotionality

As indicated elsewhere (Prus 2013a), Aristotle provides an exceptionally potent (detailed, analytically sophisticated) statement on emotionality that not only is consistent with an interactionist approach to the study of emotionality but also extends interactionist conceptualizations (e.g., Prus 1996:173–201) in distinctively enabling terms.

Defining emotions or passions as feelings or dispositions pertaining to pleasure (and pain) that have a capacity to affect people’s judgments, Aristotle intends to establish the relevancy of people’s emotions for influence work.

In this remarkable analyses of emotionality directed toward others in judicial settings (but by extension, potentially any target, including oneself, by any tactician), Aristotle deals with (1) anger and calm, (2) feelings of friendship and enmity, (3) fear and confidence, (4) shame and shamelessness, (5) kindness and inconsideration, (6) pity and indignation, and (7) envy and emulation. In addition to providing (a) instructive definitions of these emotional states, Aristotle considers (b) the foundations of these emotional states, (c) the ways that these emotions are experienced (by whom, in what ways, and with what behavioral consequences), and (d) how speakers may enter into and shape the emotional sensations, viewpoints, and actions of others.

Although Aristotle’s work on the emotionality in *Rhetoric* is much too extensive to consider in fuller detail, I have presented some of Aristotle’s materials the address people’s experiences with shame to give readers a better sense of Aristotle’s considerations of the ways that people might experience emotionality as well as shape the emotionality that others (as in adjudicators in forensic cases) might experience.

Readers familiar with Erving Goffman’s (1963) *Stigma* may appreciate just how much Aristotle has to offer in this area alone. While Goffman’s work focuses on the ways that people attempt to avoid as well as minimize disrespectability with respect to others on a more personal (i.e., as targets) level,^24^ Aristotle more directly attends to circumstances in which people are apt to experience intensified or minimized senses of shame and how speakers (as agents) may generate sensations of these sorts on the part of judges.

In attending to *Shame and Shamelessness*, Aristotle (BII, VI) defines shame as a feeling of pain or discomfort associated with things in the *present, past,* or *future* that are likely to discredit or result in a loss of one’s character.

By contrast, shamelessness or impudence is envisioned as a disregard, contempt, or indifference to matters of disrepute. Shame, according to Aristotle, revolves around things envisioned as disgraceful to oneself or to those for whom one has regard.

Among the kinds of things around which people more *commonly experience shame*, Aristotle references: (a) cowardice; (b) treating others unfairly in financial matters; (c) exhibiting excessive frugality; (d) victimizing those who are helpless; (e) taking advantage of the kindness of others; (f) begging; (g) grieving excessively over losses; (h) avoiding responsibility; (i) exhibiting vanity; (j) engaging in sexually licentious behaviors; and (k) avoiding participation in things expected of, or lacking possessions generally associated with, equals.

Further, while noting centrally that shame is *apt to be intensified* in all discreditable matters when (a) these things are deemed voluntary and, thus, *one’s fault*; Aristotle also observes that (b) people also may feel shame about dishonorable things that have been done, are presently being done, or seem likely to be *done to them by others*.

Acknowledging the anticipatory or imaginative *reactions of others*, as well as actual instances of experiencing disgrace, Aristotle subsequently *identifies the witnesses* or others in front of whom people (as targets) are apt to experience greater shame.

Most centrally, these witnesses include people whom targets hold in higher esteem (respect, honor) and admire (friendship, love) as well as those from whom they (targets) desire respect and affective regard.

People (as targets) also are likely to experience heightened senses of shame when they are disgraced in front of those who have control of things that targets desire to obtain, those whom targets view as rivals, and those whom targets view as honorable and wise.

Observing that targets are particularly susceptible to shame when dishonorable things occur in more public arenas, Aristotle also posits that people (as targets) are likely to feel greater shame when the witnesses include people who: are more innocent of things of this sort; adopt more intolerant viewpoints; and generally delight in revealing the faults of others.

Another set of witnesses or audiences in front of whom people (as targets) are more likely to experience disgrace include: those before whom [targets] have experienced success or been highly regarded; those who have not requested things of [targets]; those who recently have sought [target] friendship; and those likely to inform other people of [target] shame-related matters.

As well, Aristotle states that people (as targets) also are apt to experience shame through things associated with the activities or misfortunes of their relatives and other people with whom targets have *close connections* (i.e., experience an extension of the stigma attached to their associates).

Shame also seems intensified when people anticipate that they will remain in the presence of those who know of their losses of character. Conversely, Aristotle suggests that people are less apt to experience embarrassment among those who are thought inattentive or insensitive to such matters.

Relatedly, while Aristotle notes that people may feel comfortable with certain [otherwise questionable circumstances or practices] in front of intimates versus strangers, he also states that people (as targets) are apt to experience intensified shame among intimates with respect to things that are regarded as particularly disgraceful in those settings.

However, among those that they encounter as strangers, discredited people tend to be concerned only about more immediate matters of convention.

Aristotle ends his analysis of shame with the observation that shamelessness or the corresponding insensitivity to stigma will be known through its opposite.

Still, speaking for the entire range of emotionally oriented designations that Aristotle introduces, it should be recognized that in addition to (a) the parties being judged serving as targets, the speakers involved may (b) present themselves or their opponents as targets for various kinds of definitions, as well as (c) envision those serving as judges as yet another set of targets for their emotionally oriented definitions of self and other). Relatedly, Aristotle is entirely aware of the theatrical and dramatic nature of contested cases as well as the tentative, adjustive realism, skepticism, and affectations of people’s presentations as cases unfold as well as the ensuing realism of the eventual decisions of the judges overseeing the cases at hand.

While recognizing the potency of emotionally-oriented “definitions of the situation” for wide manners of orientations within any instance of charge and defense, Aristotle has yet much more to offer to an analysis of the deviance-making process.

## Enacted Features of Influence Work

Following his instructive analysis of emotionality, Aristotle (BII, XVIII) focuses more directly on the enacted or engaged features of persuasive activity. Briefly commenting on deliberative rhetoric, Aristotle addresses the more general construction of speeches:

The use of persuasive speech is to lead to decisions…This is so even if one is addressing a single person and urging him to do or not to do something, as when we advise a man about his conduct or try to change his views: the single person is as much your judge as if he were one of many; we may say, without qualification, that anyone is your judge whom you have to persuade. Nor does it matter whether we are arguing against an actual opponent or against a mere proposition; in the latter case we still have to use speech and overthrow the opposing arguments, and we attack these as we should attack an actual opponent…

We are now to proceed to discuss the arguments common to all oratory. All orators are bound to use the topic of the possible and impossible; and to try to show that a thing has happened, or will happen in the future. Again, the topic of size is common to all oratory; all of us have to argue that things are bigger or smaller than they seem, whether we are making deliberative speeches, speeches of eulogy or attack, or prosecuting or defending in the law-courts. (Aristotle, Rhetoric, BII, XVIII [Rhys Roberts, trans.])

Attending to the more overtly engaged aspects of rhetoric, Aristotle subsequently deals with (1) generating and refuting proofs; (2) amplifying and diminishing the images of things; and (3) arranging and deploying the components of the speech. Even here, however, readers should recognize the ways in which anticipatory, contemplative and adjustive features of speaker activities permeate the more situated features of oratorical performance and interchange. Likewise, far from “being left behind,” it should be appreciated that Aristotle is highly mindful of the emotional states that judges and other participants are apt to experience as they jointly work their ways through the entire definitional process.

### Generating and Refuting Proofs

As a means of introducing the matter of proofs (i.e., claims, arguments, cases) and challenges that speakers normally present in forensic cases, Aristotle embarks on a consideration of (1) possibilities and probabilities prior to discussing (2) the formulation of proofs and (3) their points of vulnerability for challenge. Space simply does not allow for a more extended commentary on these deviance related topics (see Aristotle’s *Rhetoric*; also Prus 2008a) but even the very sketchy discussion following may help alert readers to the exceptionally relevant and highly detailed considerations of people’s “definitions of situations” that Aristotle provides.

In addition to providing notably extended analytic considerations of *possibilities and probabilities* (BII, XIX) as this pertains to the definitions of activities, outcomes, participants, and sequences of events, Aristotle (BII, XX-XVI) deals with the matter of developing reasoned deductions, inferences, or conclusions regarding events by identifying over twenty generic tactical practices speakers may adopt in generating *proofs* for the particular positions they are representing. Relatedly, recognizing the problematic, negotiable nature of courtroom definitions, Aristotle (BII, XXV) also outlines a set of generic procedures speakers may introduce in challenging or *refuting* the proofs and claims that oppositionary speakers have presented.

In discussing the matter of *amplifying and diminishing* aspects of the images (and claims) of the things (e.g., people, objects, events, and outcomes) that have become part (focal points of various sorts) of the more immediate theater of operations in which the speakers, judges, and other participants find themselves, Aristotle (BIII, I-VI) more directly addresses modes of verbal expression.

After counseling skepticism about the value of poetic expression (wherein he deals with delivery, expressivity and audience experiences in some detail) Aristotle emphasizes clarity and authenticity in striving for more consequential sharedness of meanings, particularly in forensic and deliberative rhetoric.

Thus, Aristotle (BIII, VII-XI) deals in considerable detail about the importance of (a) the particular kinds of words and expressions that speakers use to connect with their more immediate audiences, (b) the styles of delivery appropriate to audiences, and (c) speakers’ use of metaphors in developing their cases. Aristotle (BIII, XII) subsequently compares the presentations speakers might make in spoken versus written rhetoric as well as the importance of adjusting to different sizes and contexts of audiences.

In the last sector of *Rhetoric*, Aristotle (BIII, XIII-XIX) focuses on the arrangements of the parts of a speech and the ways in which the materials in each part might be organized. He provides rationale, explanations, and considers strategic implications for the overall presentation. While observing that demonstrative oratory, because of its expressive quality, is less constrained by matters of chronological sequence, clarity, and completeness, and that forensic rhetoric normally is subject to more extensive procedural constraints, Aristotle delineates four basic parts of rhetorical presentations.

In addition to (a) the introduction (*proem* or *exordium*) which serves as the opportunity for each of the speakers to set the stage to their own advantage for the ensuing event, Aristotle also attends to the importance of speaker attentiveness to (b) the contents and styles of presentation of the *narration* (one’s account of the matter under consideration), (c) the *proofs* (claims and counterclaims) of the case, and (d) the *peroration* or concluding statements strategically directed towards the judges prior to their assessments and dispositions of the particular cases before them.

Aristotle’s analyses of the ways that people and events are defined and the ways that matters of culpability and treatment may be negotiated are exceptionally relevant to pragmatist / interactionist conceptions of the broader deviance-making process in contemporary and ongoing comparative terms.

Even though the symbolic interactionists have generated a body of highly instructive materials pertaining to the deviance-making and labeling processes (as indicated in the works of Lemert, 1951, 1962; Garfinkel 1956; Becker 1953; Goffman 1963; and Prus and Grills 2003), a great deal of pertinent insight can be gained by examining Aristotle’s works in both comparative and conceptual analytic terms.

Relatedly, although Aristotle’s *Rhetoric* does not fit more conventional notions of ethnography, it is difficult to deny its value for comprehending influence work as a realm of human activity in another place and time. Despite its specific instructional quality, Aristotle’s highly analysis of rhetoric is both comprehensive and highly detailed. More directly, Aristotle’s work is loaded with contextual insights, comparative analysis, and points of scholarly inquiry pertaining to wrongdoing, emotionality, law, and justice as processes that are steeped in influence work and resistance.

## Aristotle’s “Theory of Deviance” in Perspective

To more adequately acknowledge the substance and depth of Aristotle’s “theory of deviance,” I compare his materials with an interactionist approach using Prus and Grills’ (2003) *The Deviant Mystique* as a reference point. Providing an extended conceptually and methodologically oriented symbolic interactionist statement on the study of deviance, Prus and Grills [P&G] emphasize the necessity of approaching deviance as a community phenomenon. In the process, they envision “the deviance-making process” as taking place within an array of interactively engaged theaters of operation.^25^

Although I will only briefly introduce the P&G text, supplementing this with a more detailed table of contents in the Appendix, this along with the discussion following may be sufficient to provide a series of comparison points for appreciating the scope and enduring relevancy of Aristotle’s *Nicomachean Ethics* and *Rhetoric* for more extended examinations of deviance as interactively achieved community-based essences.

Denoting a research agenda for studying any and all realms of deviance, *The Deviant Mystique* is organized around (1) an interactionist approach to the study of deviance, (2) deviance as a community phenomenon, (3) definitions of phenomena as “deviance,” (4) definitions of persons as “deviants,” (5) people’s experiences as participants in deviance life-worlds, (6) the social organization of people’s deviance life-worlds, (7) the regulation of deviance, (8) people’s disinvolvements from deviance, and (9) an interactionist methodology for the study and analysis of deviance as participatory fields of community life.

Approaching deviance more entirely in sociological terms, P&G address deviance within the context of ongoing community life. Envisioning deviance as a matter of audience definitions and acknowledging the relative and negotiated nature of people’s (group-based) conceptions of reality, P&G first consider (a) people’s conceptions of what constitutes deviance and (b) how people (as individuals, groups and categories) become identified as deviants and the implications of these designations for their relations with others.

Next, discussing the related matter of people “experiencing deviance,” P&G attend to (a) people’s involvements in and ensuing careers of participation in various realms of deviance, (b) the nature of people’s experiences in particular subcultural life-worlds, and (c) the processes of forming, coordinating, and sustaining associations, as well as (d) the nature of people’s experiences with “solitary deviance.”

When addressing “regulating deviance,” P&G consider (a) the ways in which people deal with deviance informally and/or involve third-party others in their control endeavors, (b) the challenges of establishing, promoting, and maintaining control agencies, and (c) the ways in which people assuming roles as agents of control approach their activities, deal with particular sets of target-clientele others, and more personally come to terms with the organizational subcultures in which they operate.

Then, after attending to the processes and problematics of people’s disinvolvements from deviance, Prus and Grills conclude this volume with a discussion of the ways in which people may examine deviance as a community essence in ethnographic and comparative analytic terms.

In what follows, I first discuss the overarching affinities of the interactionist approach with the materials earlier introduced from Aristotle’s *Nicomachean Ethics* and *Rhetoric*. Next, I consider some of the major contributions that the contemporary interactionist approach makes to the study of deviance. The paper concludes with a statement on the more specific contributions of Aristotle’s *Nicomachean Ethics* and *Rhetoric* to the sociological study of deviance. First, though, there are important affinities to be acknowledged.

In the most basic terms, both Aristotle and the Chicago-style or Blumerian interactionists as represented here by P&G assume a *pragmatist* approach to the study of human knowing and acting. Focusing on “what is,” *activity* represents the central starting point for the study of human group life. Still, for both Aristotle and the interactionists, human activity encompasses much more than physical motions and physiological capacities. As a feature of ongoing community life, activity is contingent on meaningful, purposive behavior; that is behavior that is both linguistically enabled and informed through people’s active participation in the life-worlds of the community-based other (also see Prus 2007c).

Relatedly, for both Aristotle and the interactionists, phenomena do not have inherent meanings but take on meanings as people collectively (mutually) act towards reference points in more particular ways and compare these with other matters of their awareness. Relatedly, activity becomes meaningful and focused relative to the concerns or purposes that people associate with particular goals, outcomes or activities as significant reference points. It is mindful of this emphasis on activity that both Aristotle and the interactionists emphasize the importance of agency in human knowing and acting. However, it is agency, within limits, even as people make adjustments in attempts to achieve particular outcomes in the midst of the situations and resistances they encounter.

For Aristotle and the interactionists as well, activity is to be understood centrally in terms of symbolic interchange – wherein language provides the basis on which mutual indications, awareness, meanings, and understandings take shape. Still, it is in the acquisition of language and by attending to the standpoint(s) of “the community-based other” that people acquire capacities for reflectivity, deliberation or reasoning, and strategic (minded) adjustment. Both Aristotle and the interactionists take the viewpoint that humans are not born with pre-existing knowledge states or understandings, but (as instances of a *tabula rasa*) learn about the “whatness” of community life through linguistic instruction and ongoing association with others.

For both Aristotle and the interactionists, people are to be understood most fundamentally as social beings, as community-enabled essences – with the further implication that human knowing and acting cannot be achieved or understood apart from people’s participation in group life. Relatedly, as with Aristotle, the interactionists take the viewpoint that one does not require a special theory for deviance or any other realm of human endeavor. Instead, all realms of activity and all conceptions of “whatness” (what is and what is not) – that is all fields of human knowing and acting – are to be understood and examined in conceptually parallel terms.

While acknowledging the diversity (and relativism) of knowing and acting across human communities and groups within, the more central emphasis is on people’s perspectives, “definitions of situations,” and the interchanges entailed in the meaning-making process. For Aristotle and the interactionists as well, the study of human activity is to focus on instances, following the developmental, processual flows or emergence of particular events and the interchanges taking place within.

Still, it is through a comparison process (what Aristotle terms *analytic induction*) of the instances that people arrive at more basic conceptualizations of the qualities of particular sets of instances. Thus, it is in the process of attending to similarities, differences, and the implications thereof that people arrive at fuller (conceptual) understandings of the processes (and connections) by which particular kinds of events take place.^26^

In addition to attending to the interpretive or sense-making capacities of humans and people’s abilities to anticipate and adjust to one another, both Aristotle and the interactionists recognize human capacities for intentioned deception as well as focused sincerity. Whereas the interactionists have been ethnographically as well as conceptually mindful of the negotiable nature of human group life, they have become more explicitly attentive to people’s capacities for representing and misrepresenting information shared with or directed towards others through the dramatism of Kenneth Burke (1945 1950) and the dramaturgical sociology of Erving Goffman (1959, 1963). Nonetheless, as indicated in his extensive analysis of rhetoric as well as his more focused depiction of fictional productions (*Poetics*), Aristotle is acutely mindful of on community life as denoting instances of living as well as contrived and contested theater.

Notably, to their respective credit, the Blumerian interactionists, like Aristotle, have been concerned about defining their terms of reference, studying things in instances, and emphasizing the conceptual understanding and articulation of generic processes through comparative analyses (see Blumer 1969; Lofland 1976; Strauss 1993; Prus 1996, 1997, 1999; Prus and Grills 2003). As noted elsewhere (Prus 2003, 2007a, 2008a, 2009, 2013b; Puddephatt and Prus 2007), there are more affinities between Aristotle’s conceptions of community life (and deviance) and the conceptual emphases of contemporary symbolic interaction, but these may suffice for present purposes.

### Contemporary Interactionist Contributions

Given the many affinities of Aristotelian and interactionist pragmatism, I here focus on the more unique emphases and contributions of symbolic interactionism to pragmatist scholarship on a more contemporary plane. First, although the interactionists have given little attention to the Greek foundations of contemporary pragmatist social thought (especially Plato and Aristotle; Prus 2003; 2004) or ethnohistorical analyses (e.g., Thucydides’[circa 460–400 B.C.E.] *History of the Peloponnesian War*; see Prus and Burk 2010), the interactionists (most notably Herbert Blumer 1969) have developed an exceptionally coherent pragmatist and ethnographically informed approach to the study of human group life. This includes a systematic articulation of the premises or assumptions and root images undergirding the interactionist approach as well as a synthesis of pragmatist social thought and ethnographic inquiry.

Relatedly, symbolic interactionism stands in stark contrast to the structuralist and quantitative approaches that characterize so much of the prevailing social sciences as well as the moralism and activism associated with Marxism and the derivatives thereof (see Prus 1999). The interactionists (especially see Blumer 1928, 1969; Prus 1996, 1999, 2007d; Prus and Grills 2003; Grills and Prus 2008) insist that the study of human knowing and acting requires a very different conception of science than that used to study physical phenomena.

What is required is an approach that not only attends to the fundamentally group-based, linguistically-enabled nature of human knowing and acting but that also recognizes people’s capacities for interpretation, intentioned, purposive behavior, and strategic, adjustive interchange. Further, and despite the commonplace tendencies in the social sciences to reduce the study of human knowing and acting to individual qualities (including inborn physiological or internalized psychological states or dispositions), the interactionists have maintained a clear emphasis on the centrality of human group life for comprehending all meaningful realms of human knowing and acting.

Focusing on the ways in which people make sense of and participate in situations in collective as well as in individual terms, interactionist analyses are strikingly sociological (versus psychological) in emphasis. Notably, thus, the interactionists (like the ethnographically oriented ethnomethodologists and social constructionists) have focused on the linguistic and activity oriented, collectively achieved foundations of human group life – attending to the ways that human group life is accomplished in instances on a day to day, moment to moment basis. Although the interactionist viewpoint on these matters very much resonates with Aristotelian pragmatism, it is the interactionists (and the kindred scholars just referenced) who have given these matters a distinctively enabling conceptual and methodological (i.e., empirical) prominence in the contemporary social sciences.

Whereas the interactionists have introduced a particularly valuable array of “terms of reference” for conceptualizing the study of human group life,^27^ they also have methodologically and conceptually extended the ethnographic examination of community life. Indeed, more than any other group of historical record, the interactionists have compiled a century of relatively conceptually coherent, pragmatist oriented ethnographic materials (see Prus 1996, 1997, 1999).

To a very large extent as well, these materials have focused on “what is” from the viewpoint of the participants rather than providing prescriptions or moralizations about what people should do. Further, attending to Herbert Blumer’s insistence on the importance of generic, process-oriented concepts, the interactionists have sought to develop ethnographically informed conceptual material through the use of more sustained comparative analyses (e.g., see Lofland 1976; Strauss 1993; Prus 1996, 1997, 1999; Prus and Grills 2003). The further implication is that each subsequent study could provide opportunities to more fully examine, “test out,” assess, and revise current conceptualizations of human knowing and acting.

As a result, the interactionists have accumulated an exceptional corpus of ethnographic research that is characterized by the quest to achieve “intimate familiarity with one’s human subject matter” (Blumer 1969). While this literature could be subjected to more sustained comparative analysis, the potential of this endeavor is particularly evident in the materials developed around people’s involvements and longer-term participation in subcultural life-worlds (deviant and otherwise) as well as theory on the social organization of the subcultural life-worlds that people develop as they go about their activities and interchanges in more particular realms of association (e.g., Prus 1996, 1997, 1999).

Benefiting from a thorough embeddedness in the interactionist tradition, the P&G text more specifically represents a systematic and focused agenda for studying deviance as a realm of human lived experience. As such, *The Deviant Mystique* synthesizes interactionist theory, methodology, and existing ethnographic research in formulating a series of generic social processes amenable to subsequent inquiry of the deviance-making process.

Thus, the P&G text provides more focused considerations of (a) the processes by which people define “what is deviance” and “who the deviants are,” relative to audience conceptions of morality and disrespectability; (b) people’s careers of participation (initial involvements, continuities, disinvolvements, and re-involvements) and associated interchanges with others take place; (c) the ways in which people organize their subcultural life-worlds and how they coordinate the collective events that take place therein; and (d) the social life-worlds that develop around people’s attempts to regulate deviance – as well as deal with the many potential points of resistance that the regulation of deviance may entail (also see Blumer 1971).

Relatedly, P&G take the deviance phenomena apart, piece by piece, and discuss these processes in more distinctively emergent terms. Not only do P&G (a) attend to people’s activities within the settings at hand and (b) focus on the ways in which people work out their activities in conjunction with others in those contexts, but they also do so (c) in ways that are mindful of the fuller sets of participants in people’s theaters of operation.

Systematically addressing deviance as a set of interrelated, community-based processes, *The Deviant Mystique* represents a *research agenda* for enabling conceptually focused ethnographic research and comparative analysis in any realm of human endeavor that some audience might define as disreputable. The approach is non-prescriptive and is intended to provide opportunities to consider openly (and nonjudgmentally) the ways that all participants in particular theaters of operation become involved, experience, and deal with others in the activities and settings under consideration. The objective is to understand the deviance-making process in the fullest humanly-engaged, humanly experienced terms possible by focusing more entirely on “what is.”

The broader interactionist implication is that one not only would learn about deviance through ethnographic research and comparative analysis of people’s involvements in other realms of community life but that one also would learn more about people’s involvements in other realms of community life by examining in more sustained detail the nature of people’s involvements in the deviance-making process.

Attending to human group life “in the making” in highly sustained conceptual and methodological terms, the contemporary interactionists and kindred scholars have a great deal to offer to the enduring study of deviance as a community-based process.^28^ Still, from an interactionist standpoint, there is considerably more to be gained from an extended examination and synthesis of the materials that Aristotle has provided.

### Aristotle’s Contributions to the Study of Deviance

Having presented a partial and compacted but still fairly substantial, “chapter and verse,” overview of *Nicomachean Ethics* and *Rhetoric* to help illustrate the remarkable scope of Aristotle’s pragmatist analysis of the human condition, I briefly identify yet more of the conceptually enabling qualities of Aristotle’s works.

Emphasizing the centrality of *activity* for comprehending the human condition, Aristotle also makes the point that while people’s activities are primarily valued for what they could achieve (i.e., outcomes, goals, objectives), people’s particular activities become focused and are invoked because of people’s intended and anticipated ends. Still, insofar as activities may be valued for what they can help people attain, people’s definitions of activities and the ways these are pursued and developed are best understood in reference to other aspects of ongoing community life.

Thus, Aristotle emphasizes the fundamentally enabling nature of the community – for it is *the living, activity-based community* that most consequentially sustains human existence and gives meaning to all fields, realms, and instances of endeavor. Relatedly, not only is the community greater and more important than the particular individuals within but *all instances of knowing and acting* are to be comprehended within the interchanges and interconnected contexts of human group life.^29^

Although this attentiveness to the centrally enabling qualities of the community is less explicit in American pragmatist thought and its sociological derivative, symbolic interactionism, Aristotle’s emphasis on the centrality of the community for comprehending all meaningful instances of human knowing and acting stands in sharp contrast to the relatively pervasive emphasis on the individual in contemporary psychology and the great many associated individually-based explanations of deviance developed thereof.

Aristotle’s insistence on the importance of the enacted features of community life also stands in distinct contrast to those sociologists and political scientists who try to predict and control people’s “behavioral outcomes” by emphasizing (essentially dehumanized) structural / factors-oriented approaches. For Aristotle, “the science of the polis” represents the study of *the enacted social organization of the community*. In addition to (a) governing practices, confrontations, and transitions therein, this includes (b) the relationship of the community to the individuals within, and (c) the relationships of the individuals within the community relative to one another.

Relatedly, it is within the context of ongoing community life that notably consequential conceptions of morality, deviance, justice, and regulation are developed, promoted, enforced, sustained, challenged, and possibly reshaped. Still, for Aristotle, all aspects of “the deviance-making process” (like all other realms of knowing and acting) are to be understood in essentially parallel, humanly engaged terms – even though the substantive contexts, their evaluations, and the particular individuals involved can vary greatly.

Whereas Aristotle maintains a pronounced emphasis on the centrality of community life and people’s activities within, he also is mindful of (a) people’s individual experiences and participation as social essences within the various community contexts in which they find themselves as well as (b) the dependency of the community on the activities and interchanges of the people within for the overall sense of harmony and direction of the community. Accordingly, Aristotle is acutely attentive to the enacted relationship of “the individual” to an array of community-based others. Although aspects of these interdependencies are evident in George Herbert Mead’s (1934) *Mind, Self and Society* as well as Herbert Blumer’s (1969) *Symbolic Interactionism* and especially Blumer’s (1971) “Social Problems as Collective Behavior,” Aristotle pursues this matter in more comprehensive, analytically enabling terms.

Aristotle likens humans to other animals in that humans have capacities for sensation and motion as well as variable states of organic tension. However, he clearly envisions humans as animals that are to be understood within the context and parameters of a linguistically-enabled community life. Even though people may develop more individualized habits at preverbal and then linguistically interfused character levels, Aristotle is mindful of the instruction (however uneven this may be) that humans receive from others regarding “the whatness” of community life.

It is as linguistically-enabled beings that humans develop (a) capacities for minded awareness, reasoning, agency, and wide ranges of voluntary activity as well as (b) conceptual frames for assessing self and other and (c) tactical orientations for regulating others as well as themselves. It is through people’s participation in the various community-based theaters of others that the more specific, as well as the more encompassing, meaningful nature of people’s lived experiences take shape.

Although Aristotle, at times, seems intent on promoting more virtuous or honorable (personally and interpersonally) modes of human knowing and acting for the benefit of the community and the people within, his analyses of *habits* and *character* have very fundamental, cross-contextual qualities that could serve to extend interactionist (and other social science) conceptions of people’s senses of self and other. Indeed, more than the interactionists (and other contemporary social scientists), Aristotle addresses *character* as a developmental, meaningful, interactively accomplished and reflectively engaged process.^30^ Quite directly, the study of character as a sociological phenomenon adds a valuable dimension of “tentative continuity” to the more general interactionist tendency to focus on the more situated aspects of the instances at hand.

While his conception of character in *Nicomachean Ethics* has a generic emphasis that extends well beyond considerations of deviance, Aristotle’s analysis of character could add substantially to interactionist conceptions of people’s identities, reputations, and interchanges. Because Aristotle approaches character in activity-based and reflective process-related terms, his work also could significantly advance interactionist studies of the stabilization and transformation of people’s activities and involvements in the community at large and the study of deviance and regulation more specifically.

Accordingly, thus, Aristotle’s conceptions of self-regulation, wisdom, reasoning practices, and voluntary activities represent particularly potent points of departure for interactionist inquiry as also do his distinctions between preverbal habits and linguistically-enabled virtues in *Nicomachean Ethics*.

Moreover, whereas most contemporary scholarship has focused on people “doing deviance” (to the relative neglect of “doing good”), Aristotle explicitly recognizes the interrelatedness of these two (morally differentiated) realms of activity and the importance of studying each (and people’s definitions thereof) relative to the other.

Aristotle also is highly cognizant of the problematic matter of self-regulation – particularly amidst the challenges that people face in making choices when they encounter more ambiguous (especially dilemma-related) instances. Relatedly, Aristotle’s work on emotionality (in *Rhetoric*) and the associated matter of people attempting to shape the affective viewpoints and activities of others as well as their own emotions and practices (Prus 2013b) represents an exceptionally valuable set of departure points for the study of self (and other) regulation. Whereas the interactionists have given some attention to emotionality as a socially engaged process (Prus 1996: 173–201), there is much to be gained from a closer study of Aristotle’s analyses of emotionality as a socially engaged process.

Still, another very consequential point of mutuality and an associated extension of interactionist scholarship should be noted. This revolves around the interactionist emphasis on the negotiated nature of reality and their attentiveness to human interchange in much of their ethnographic inquiry. Although not presented as “an instance of ethnography,” Aristotle’s *Rhetoric* represents one of the most detailed, substantively informed and conceptually articulated accounts of persuasive interchange and impression management that exists in the literature.^31^ This text also offers a valuable set of reference points for considering tactical interchange in the judicial processing of deviance (see Garfinkel 1956 for a more limited but still insightful analysis of “the conditions of successful degradation ceremonies”).

Further, whereas Aristotle acknowledges the generic nature of the influence process across the entire scope of community life, *Rhetoric* adds substantially to the entire process of explaining the deviance-making process – including the matters of anticipated, planned, focused, enacted, interactive, contested, intendedly deceptive, and theatrically shaped features of judicial interchange as well as issues pertaining to people’s conceptions of justice, moral indignation, culpability, perpetrator-oriented sympathy, and victim compensation.

Notably, too, Aristotle’s attentiveness to *causality* as a humanly engaged (purposive, contemplated, directed, enacted, observed, defined, and contested) process helps illustrate the intellectual poverty of structuralist (and factors-oriented) conceptions of causality for explaining deviance as well as other realms of human knowing and acting (also see Blumer 1969; Prus 1996, 1999, 2010; Prus and Grills 2003; Puddephatt and Prus 2007; Grills and Prus 2008).

While some may still insist that “the latest is the greatest” and yet others may deny the relevance of newer developments saying that “there is nothing new under the sun,” it can be highly productive for students of human group life to examine materials from the past as well as the present in more sustained comparative terms – especially when these are developed in more extended conceptual and ethnohistorical terms.

When this is done in the case of contemporary symbolic interactionism and Aristotle’s contributions to the study of human knowing and acting, the benefits of a more sustained synthesis (as briefly suggested herein) can be substantial indeed. Still, in looking ahead, there are some other matters that present day and future students of the human condition might acknowledge.

First, the interactionist tradition discussed herein reflects a century of focused scholarship as well as a current, actively engaged realm of study. Whereas contemporary interactionist scholarship may have taken root in North America, interactionism more recently has been acquiring a more extended presence in Europe, along with some extensions in Asia. Still, what is much more consequential is the remarkable array of concepts, methodological resources, sustained studies of people’s lived experiences, and the comparative analytic standpoints that presently are available to those pursuing the study of “the whatness of human group life” in more open and extended terms.

Moreover, because of its multiplistic but also non-moralistic humanly engaged approach to the study of community life, Chicago-style interactionism has an exceptional transcontextual and transhistorical relevance. As such, this approach, its literary resources, and the dedicated community of scholars within represent particularly consequential centering points for better understanding, studying, and conceptualizing human group life – within and across all societies, all life-world contexts therein, and all instances of human knowing and acting – in the present as well as across all literary accessible time frames.

Aristotle may have lived nearly 2500 years ago and his (not so explicitly designated) pragmatist scholarship may seem less obvious than that of the contemporary symbolic interactionists. However, it should be recognized that (as an integral component of classical Greek thought) Aristotle’s texts have achieved a distinctively valuable (albeit primarily Western) “worldly presence” – even amidst the broad array of historical transformations and the ravages of natural events that have taken place over the last two millennia. Indeed, a scholarly awareness of and attentiveness to Aristotle’s emphasis on community, activity, and interchange have been far from continuous.

Nevertheless, there is a general informed receptivity to the exceptionally enabling potency of classical Greek scholarship across and beyond Europe. Thus, even though substantial aspects of Aristotle’s scholarship have been lost, distorted, and ignored over the millennia, the more enduring legacy of Aristotle’s scholarship signifies a fundamental as well as consequential base for more fully extending the study of human knowing and acting in pragmatist and ethnographic terms.

However, in addressing the more immediate present as well as the upcoming future we are dependent on interactionist-oriented and like-minded scholars (a) “reaching back in time” and building on Aristotle’s exceptionally generic, “forward reaching” scholarship as well as (b) searching for, attending to, and conceptually comparing parallel instances of interim pragmatist and ethnohistorical literary statements on human knowing and acting.^32^

Thus, as we look toward the ever unfolding future, contemporary interactionists and kindred scholars are in an integral position to build on a much more extended set of conceptual and ethnohistorical materials – for learning more about the variations in human lived experience and, no less instructively, using these as resources for sustained, conceptually-oriented comparative analyses.

Whereas Western scholarship has been set back numerous times and in various ways as a consequence of broad ranges of political, religious, and moral agendas (and associated scholarly disregard and hostilities) as well as natural disasters, it is most important that we maintain an emphasis on *the study of community life as an enacted realm of human lived experience*. As history teaches us (also see Durkheim’s [1904–1905] *The Evolution of Educational Thought*), more specialized realms of scholarship, as well as even the seemingly most fundamental scholarly emphases, have fragile existences. We may be unable to determine the ways in which future generations might pursue academic ventures, but we still have an obligation to extend, as much as we can, interactionist and other pluralist-oriented ethnographic approaches to the study of human lived experience.

## Appendix

**The Deviant Mystique: Involvements, Subcultural Realities, and Regulation**

Robert Prus and Scott Grills

The Conceptual Frame

1. Encountering the Deviant Mystique: Fascination, Indignation, and the Dramatization of Evil
  • Framing the Analysis
  • Acknowledging the Deviant Mystique
  • Permeating the Deviant Mystique
2. Intersubjective Accomplishment: Human Knowing and Acting
  • Attending to Human Lived Experience
  • Symbolic Interactionism
  • Ethnographic Research: Questing for Intimate Familiarity
3. Theaters of Operation: Deviance as Community Enterprise
  • Experiencing Deviance
  • Managing Trouble
  • Participating in Public Forums
  Designating Deviance
4. Defining Deviance: Perspectives and Practices
  • Moral Entrepreneurs
  • Social Problems as Collective Behavior
  • Cultures of Social Problems
  • Toys and Other Fictionalizations
  • Striving for Success
  • Resisting Moral Impositions
5. Labeling Deviants: Disrespectable Persons
  • Labeling Theory
  • Constructing Social Identities
  • Stabilizing Identities and Involvements
  • Dealing with Disrespectability
  Experiencing Deviance
6. Becoming Involved: Subcultural Mosaics and Careers of Participation
  • Subculture as an Analytic Concept
  • Subcultures and Subcultural Mosaics
  • Careers of Participation
  • Initial Involvements, Continuities, Disinvolvements, and Reinvolvements
7. Engaging Subcultures: Interactive Life-Worlds
  • Acquiring Perspectives
  • Achieving Identity
  • Doing Activity
  • Making Commitments
  • Managing Relationships
  • Developing Linguistic Fluency
  • Experiencing Emotionality
  • Participating in Collective Events
8. Subcultural Ventures: Forming and Coordinating Associations
  • The Grouping Process
  • Establishing Associations
  • Objectifying Associations
  • Encountering Outsiders
9. Solitary Deviance: Alone with Others
  • Acknowledging Solitary Activity
  • Qualifying Solitary Deviance
  • Embarking on Solitary Pursuits
  • Relating to Others
  • Conceptual Problematics
  Regulating Deviance
10. Encountering Trouble: Handling Deviance Informally
  • Dealing with Trouble
  • Defining Situations as Troublesome
  • Responding to Deviance
  • Contending with Target Associates
11. Organizational Agendas: Maintaining Control Agencies
  • Achieving Support
  • Accessing Cases
  • Classifying Cases
  • Emphasizing Treatment
  • Restoring Justice
  • Pursuing Internal Order
12. Assuming Office: Control Agents at Work
  • Acknowledging Organizational Objectives
  • Learning the Ropes
  • Maintaining Order
  • Engaging Targets
  • Sustaining Presence
13. Experiencing Disinvolvement: The Problematics of Disengagement
  • Involvement Processes and Contexts
  • Disinvolvement Processes
  • Experiencing Treatment
  • Disinvolvement, Ambivalence and Reinvolvement
  In Perspective
14. Studying Deviance: Ethnographic Examinations of Community Life
  • Transcending the Deviant Mystique
  • Examining Deviance in the Making
  • Extending the Conceptual Frame

## Footnotes

1 Beyond Blumer’s (1933) and Blumer and Hauser’s (1933) studies of adolescent experiences with the movies, other early works in this tradition include Anderson’s (1923) *The Hobo*, Shaw’s (1930) *The Jack Roller*, Waller’s (1930) *The Old Love and the New*, Cressey’s (1932) *The Taxi-Dance Hall*, and Sutherland’s (1937) *The Professional Thief*. One may note the close linkage of deviance motifs in these works with what would become known as symbolic interaction, a term Blumer introduced in 1937. While the subsequent interactionist literature on deviance is much too extensive to list here, readers may refer to the collected papers found in Herman (1995), Adler and Adler (2000), and Rubington and Weinberg (2002) as well as the many sources cited in a more sustained interactionist analysis of deviance (Prus and Grills 2003).

2 Although Plato develops two model societies in *Republic* and *Laws*, wherein the emphases are on rule by philosopher-kings and regulation by legislation, respectively, it is most inappropriate to dismiss Plato as a utopian philosopher. Not only is Plato much more mindful of the practical limitations of policy (also reform and revolutionary proposals) than are most who have followed him, but Plato also builds astutely on his knowledge of the day to day operations of various socialist, democratic, and autocratic Greek city states.

3 Those who examine *Republic* and *Laws* will find that Plato is highly mindful of many pragmatist (i.e., humanly known, enacted, contested) features of human group life – even as he promotes particular modes and realms of social organization, moral order, day to day practices, and individual virtues.

4 Plato (*Republic*) also notes that those invoking dialectic reasoning sometimes disregard the viability of everything, including notions of community morality and deviance. To offset this tendency toward philosophic cynicism, Plato insists that those training to be philosophers spend 15 years in outside pursuits to acquire a more appropriate, practical knowledge of the world before they are fit to teach philosophy to others.

5 A fuller appreciation of Plato's pragmatist approach to the matters of human knowing and acting can be found in his considerations of poetic expressions (Prus 2009), love and friendship (Prus and Camara 2010), education and scholarship (Prus 2011a), morality, deviance and regulation (Prus 2011b), and religion (Prus 2013c).

6 Those who are not familiar with Plato’s texts may be surprised to see his scathing criticisms of sophists (philosophers and educators of nonSocratic sorts), rhetoricians, and poets (see, for instance, *Georgias*, *Protagoras*, *Republic*). Plato leaves no doubt about his disaffection with those (deviants) who do not subscribe to the moral philosophy and theology associated with Socrates and Pythagoras. Many later theologians and moralists are apt to have found Plato’s invectives of considerable value in denouncing those whom they opposed on similar grounds.

7 As such, the position taken here contrasts notably with many theologians who not only have attempted to synthesize the philosophies of Plato and Aristotle but also have endeavored to do so within the parameters of particular religious (especially Christian and Islamic) frameworks.

8 Although Thomas Aquinas (c1225-1274; *Summa Theologica)* would later invoke Aristotle to buttress his version of Catholic theology, Aquinas effectively denies the (Platonic, Judaic, Christian, and Islamic) conception of an inborn spiritual soul in order to accommodate Aristotle. Following Aristotle whom he designates “The Philosopher,” Aquinas insists that people are born animals and (like other living entities) possess life-energies and biological capacities to respond to sensation. In the case of people, the term *psyche* (*de anima*) for Aquinas refers to mindedness, a life-energy or the Christian (spiritual) soul, depending on usage. Likewise, for Aquinas, following Aristotle, *psyche* or *de anima* humanly refers to an organically-achieved, activity-based, linguistically-enabled, developmentally-engaged, and community-sustained process. As a theologian, Aquinas offers no incontrovertible proof of an afterlife, but instead maintains “on faith” that those who have lived in accord with Christian precepts will be received into a divinely-enabled afterlife. It is here that Aquinas parts company with Aristotle (who makes no claims about a divinely enabled afterlife or people’s *psyches* as spiritual essences). It is here that Aquinas maintains a more distinctively theological Christian viewpoint.

9 See Aristotle’s *Politics* and *Nicomachean Ethics* (also see *On the Soul*; *Sense and Sensibilia;* and *On Memory*).

10 Somewhat ironically, Aristotle not only conceptualizes causation in terms that are much more sophisticated than those invoked in contemporary quantitative (and positivist) social science, but Aristotle clearly attends to a pragmatist or humanly engaged conceptualization of causation.

11 While especially indebted to the translations of *Nicomachean Ethics* provided by Harris Rackham and W.D. Ross, I also have benefited from the translations of *Eudemian Ethics* by J. Solomon, and *Magna Moralia* by G. Stock as well as Thomas Aquinas’ extended *Commentary on the Nicomachean Ethics*. Although there is much overlap among Aristotle’s three texts on ethics, this statement builds primarily on *Nicomachean Ethics* (the most widely accessible of Aristotle’s texts on ethics).

12 Although we have no sustained commentary on Aristotle from G.H. Mead (1934), the references Mead makes to Aristotle suggest that Mead’s direct familiarity with Aristotle’s works is rather limited. Thus, while Mead criticizes Aristotle for not appreciating the evolutionary process that Darwin develops, Aristotle anticipates Darwin’s theory of evolution in noteworthy respects (as in process and change of all known things, selective plant and animal breeding, and the significance of embryonic disruptions for later development). As well, Aristotle is highly attentive to process, activity, speech, and reflectivity in ways that resonate directly with Mead’s most basic tenets of *Mind, Self, and Society.*

13 The version of Aristotle’s *Politics* to which scholars presently have access is a rather uneven and incomplete statement. It appears to contain an assortment of subtexts that vary in emphasis and style. See Aristotle’s *Rhetoric*, and *Rhetoric to Alexander* for other materials pertaining to political science as a realm of humanly engaged endeavor.

14 In addition to discussing virtues and vices as (a) *character-based* (developmentally acquired habits, emotionalities, preferences) and as (b) *intellectually* achieved (through instruction, study, and practice), Aristotle also discusses virtues as (c) *enacted* (as with people acting in minded terms; i.e., as knowing, deliberating, choosing, monitoring, adjusting agents), (d) subject to *judgment* by others (as in responsibility, and praise or blame), and as matters of (e) *collective* as well individual concern. Unfortunately, these latter three matters and the related shifts of emphases are not always explicit in his text. Defining moral virtues as habitual tendencies or dispositions to do good (as in noble, balanced, and just manners), Aristotle characterizes people’s (prelinguistic habits) moral virtues as irrational (nonrational) essences because these emotive tendencies are physiologically and behaviorally developed. As a result, they are not as amenable to choice and direct control as are the intellectual virtues. Nevertheless, Aristotle indicates that people’s moral (character) virtues may be modified over time by mindful self-reflection as well as effectively redirected through more sustained instances of choice. That is, while virtues and vices both represent inclinations to act in certain respects, people may not only adjust their (character) dispositions somewhat over time but also more consciously deal with these dispositions when they are deciding how to act or do things.

15 While the interactionists have tended to approach human behavior in more situated terms and have been skeptical of the notion of “personality” or character as developed by those in psychology and anthropology, Aristotle’s position is notably different from these latter standpoints. Instead, Aristotle discusses people’s habitual tendencies towards virtue and vice as a context for comprehending knowingly purposive, deliberative activity and interchange.

16 Aristotle is clearly attentive to people’s capacities to attend to sensations, but he does not subscribe to the highly behavioristic notions that typify conditioning theory in contemporary psychology. As his discussion of happiness (throughout and more specifically in Book X) indicates, pleasure does not consist of, or reside in, a sensation, an action, or a definition but is a realm of experience that requires the integrated presence of all three of these elements (also see Becker 1953 account of becoming a marijuana user). Relatedly, people's definitions of pleasurable and painful experiences could shift over time and in the presence of differing audiences of others.

17 As he addresses virtues and vices, Aristotle appears to recognize multiple forms of learning (as in explicit preverbal childhood conditioning on the part of parents; the inadvertent development of repetitive styles of doing things; other kinds of sensory-enabled associational learning; explicit linguistic instruction; the knowing development of technique; applying general knowledge to specific situations; and developing and utilizing various modes of reasoning in more expansive manners). It is apparent, as well, that these various methods of learning become intermixed (i.e., less discernable) as people do things in more knowing meaningful terms. Thus, while Aristotle will later concentrate on the *intellectual virtues* as matters to which people should strive in a more noble and enabling sense, the division of virtues into these two categories (moral and intellectual) introduces some analytic difficulties.

18 Aristotle suggests that because of the virtues and vices that people develop as characters (i.e., habits, dispositions, preferences), people would not be able to control or direct their behaviors as fully as they (or others) might like. Given that characters (once established, even in more tentative terms) imply certain tendencies on the part of people, Aristotle takes the position that it would be more pleasurable for people to act in line with their existing dispositions and, conversely, more painful (if not generally more difficult or demanding) for people to act in ways that are contrary to their dispositions.

19 While building primarily on Rhys Roberts’ and J. H. Freese’s translations of Aristotle’s *Rhetoric*, this statement also benefits from Buckley’s translation of Aristotle’s *Treatise on Rhetoric*. Interestingly, although Thomas Hobbes is highly critical of Rhetoricians, Hobbes’ (1681) synopsis of Aristotle’s *Rhetoric* also was found instructive (in comparative terms) in developing the present statement.

20 As is the case of virtually all rhetoricians who have followed him, Aristotle does not give sustained attention to how audiences (or judges) actually interpret these statements. Still, Aristotle is quite aware of this process and encourages his speakers to take their audiences into account in both more generalized (as in interests, dispositions, backgrounds) as well as more engaged, situated, adjustive manners. Likewise, the materials he introduces in Books II and III of *Rhetoric* are developed with sustained reference to speakers presenting things in ways that judges would find more credible, more compelling in making their decisions.

21 Building on Aristotle and some other interim sources, Marcus Tullius Cicero (c106-43BCE) provides the next outstanding philosophic consideration of rhetoric (several texts, including *Brutus*, *De Inventione, Orator, Topics*; Prus 2010). However, after Cicero, subsequent treatments of rhetoric generally have failed to sustain the comparative-analytic qualities that Aristotle introduces in *Rhetoric*. With philosophy and rhetoric becoming more distant disciplines, the ensuing emphases in rhetoric to the present day, thus, have been more applied, technical, grammatical, and expressive.

22 Although we have no preserved legal codes from the classic Greek era (circa 700-300B.C.E.), it is quite apparent (e.g., see Plato’s *Republic* and *Laws*, as well as Aristotle’s *Rhetoric*, *Nicomachean Ethics*, *Politics*, and *The Athenian Constitution*) that the Greeks of Plato’s and Aristotle’s time were attentive to a great many civil, state, political, religious, and legal technicalities. Also see Harris (1994) on Greek law and rhetoric.

23 Readers familiar with Harold Garfinkel’s (1956) statement on “degradation ceremonies” may be struck by the conceptual similarities of Garfinkel’s analysis with the much more elaborate treatment provided by Aristotle. Still, Garfinkel’s statement was informed by the dramatism of Kenneth Burke (1945, 1950) who in turn had significantly built on (but still only very incompletely represented) Aristotle’s (much more conceptually developed) *Rhetoric*.

24 Whereas much of Erving Goffman’s “dramaturgical sociology” (1959, 1963) reflects the “dramatism” of Kenneth Burke, it should be noted that Burke (*A Grammar of Motives, A Rhetoric of Motives*) built notably – although only partially on the much more encompassing array of conceptual materials found in Aristotle’s *Rhetoric*.

25 Prus and Grills (2003) build on Mead (1934), Goffman (1959, 1963), Blumer (1969, 1971), Becker (1963), Lofland (1976), Strauss (1993), Prus (1996, 1997, 1999) and the vast array of Chicago-style ethnographic research (for an earlier but still extended review, see Prus 1997). The Prus and Grills text also benefits from two extended ethnographic examinations of the life-worlds of hustlers and thieves (Prus and Sharper 1977; Prus and Irini 1980). Very much, thus, P&G volume represents what may be termed “Blumerian” or “Chicago-style” symbolic interactionism. For a fuller range of the approaches presently falling within the broader interactionist paradigm, see Reynolds and Herman (2003).

26 Whereas Blumer (1969) has long emphasized the centrality of comparative analysis for the study and analysis of human knowing and acting, it is this process of analytic induction that Glaser and Strauss (1967) have defined as central to the development of “grounded theory” in sociological analysis.

27 In the briefest of terms, it should be noted that the contemporary interactionists have built on the pragmatist-oriented works of John Dewey, George Herbert Mead, Charles Horton Cooley, and Herbert Blumer, along with the dramatism of Kenneth Burke (1945, 1950), the dramaturgical sociology of Erving Goffman (1959), and the phenomenological sociology of Alfred Schutz (via the social constructionism of Berger and Luckmann 1966 and the ethnomethodological work of Garfinkel 1967).

28 By “kindred scholars,” I refer most specifically to ethnographically-oriented ethnomethodologists and social constructionists, along with those “realist” anthropologists who also concentrate on developing pragmatist-oriented ethnographic studies of human knowing and acting.

29 Thus, whereas a great many contemporary social scientists (in sociology and especially psychology) approach deviance, crime, and the like as “behavioral outcomes” (i.e., dependent variables) to be explained as matters unto themselves, Plato and Aristotle are much more attentive to *the interacted (activity-based) connectedness* of morality, wrongdoing, rhetoric, religion, fiction, education, family relations, and the like, as well as the notably integrated developmental flows of community life (see Plato’s *Republic* and *Laws*; also Prus 2007a, 2008a, 2009, 2011a, 2011b, 2013c).

30 For a contemporary instance of research along these lines, see Arthur McLuhan’s (2014) Aristotelian informed ethnographic study of character as a social process in two religious clergy training programs.

31 Indeed, only Marcus Tullius Cicero (circa 106–43 B.C.E.), who builds centrally on Aristotle’s *Rhetoric* along with an extended array of interim Greek and Latin sources, further extends the analysis of rhetoric as persuasive interchange and impression management. For an overview of *Cicero’s* analytic texts on rhetoric (especially *Brutus*, *De Inventione*, *De Oratore*, *Orator*, and *Topica*), see Prus (2010).

32 Aristotle has much more to offer interactionists and other students of the human condition but it may be instructive to at least briefly draw attention to the exceptionally comprehensive, analytic and investigative stance Aristotle takes to the various subject matters he addresses. Focusing on “what is,” Aristotle seems intent on taking everything apart, piece by piece, attending to the qualities and the connections of all matters that subsequently become apparent within – as these might take shape in process terms and might be examined as instances. Observations of instances are then compared (via analytic induction), asking about similarities and differences as well as changes and continuities, attending to the conceptual implications thereof. Thus, whereas Aristotle may be well known in philosophy for deductive logic (particularly as expressed in syllogistic terms), his more discerning scientific approach to the extended study of instances and sustained emphasis on comparative analyses (i.e., analytic induction) therein represents his much more consequential conceptually-enabling legacy. In addition to the exceptionally potent analyses of human knowing and acting that he provides in the texts to which we have access, students of the human community may benefit immeasurably by attending to Aristotle’s “mode of scholarship.”
